# Recent Advances in Lanthanide Complexes in Biological Systems: Coordination Principles and Interactions with Biomolecules

**DOI:** 10.3390/ijms27031566

**Published:** 2026-02-05

**Authors:** Michele Costanzo, Sabrina Bianco, Marta Fik-Jaskółka, Giovanni N. Roviello

**Affiliations:** 1 Department of Molecular Medicine and Medical Biotechnology, University of Naples Federico II, 80131 Naples, Italy; michele.constanzo@unina.it (M.C.);; 2 CEINGE–Biotecnologie Avanzate Franco Salvatore, 80145 Naples, Italy; 3 Faculty of Chemistry, Adam Mickiewicz University Poznań, 61-614 Poznań, Poland; 4 Institute of Biostructures and Bioimaging, Italian National Research Council, 80131 Naples, Italy

**Keywords:** lanthanide, complex, natural compounds, coordination chemistry, biomolecular interactions, bioimaging, bioinorganic chemistry

## Abstract

Lanthanide ions and their complexes have emerged as versatile tools in biology and medicine owing to their unique photophysical, magnetic, and coordination properties. Their applications span bioimaging, sensing, therapy and diagnostics, underpinned by their strong preference for oxygen-donor ligands, kinetic stability, and tunable luminescence. This review integrates current developments in lanthanide coordination chemistry, focusing on the mechanistic basis of their interactions with biomolecules such as nucleic acids, proteins, and peptides. Moreover, this work highlights the design principles governing complex stability and biological compatibility, summarizing key biomedical uses of lanthanides ranging from imaging and drug delivery to anticancer and antioxidant effects, and discusses their toxicity and biodistribution, and their potential for clinical translation. In particular, this review offers a mechanistically oriented synthesis of recent advances, emphasizing the interplay between coordination behavior and biological function, and identifying emerging trends that define the current landscape of lanthanide-based bioinorganic research. By correlating molecular coordination features with biological performance, the review identifies the main trends shaping lanthanide-based bioinorganic research, also including a brief discussion of complexes formed between lanthanides and naturally occurring molecules, such as amino acids.

## 1. Introduction

Lanthanide ions and their coordination complexes [1,2,3,4,5] occupy a unique position at the interface of inorganic chemistry, biology, and medicine due to their distinctive electronic structure, coordination behavior, and physicochemical properties. The trivalent lanthanides (Ln^3+^), spanning from lanthanum to lutetium, are characterized by shielded 4f orbitals, high charge density, and a strong preference for oxygen-donor ligands, resulting in predominantly ionic bonding and flexible coordination geometries. These features underpin their exceptional photophysical and magnetic properties, including sharp line-like luminescence, long excited-state lifetimes, large Stokes shifts, and strong paramagnetism, which are not accessible with transition-metal ions or organic fluorophores [6].

In biological contexts, lanthanides display a remarkable ability to mimic calcium owing to their comparable ionic radii, while simultaneously introducing enhanced Lewis acidity and coordination strength. This duality enables lanthanides to interact selectively with biomolecular targets such as nucleic acids, peptides, and proteins, often occupying Ca^2+^-binding sites while conferring spectroscopic or magnetic readouts [7,8]. At the same time, the lanthanide contraction systematically modulates ionic size, hydration number, and binding affinity across the series, providing a powerful handle for tuning biological activity and coordination stability [9].

Over the past decade, lanthanide coordination chemistry has expanded well beyond classical bioinorganic models to encompass sophisticated molecular complexes, supramolecular assemblies, metal–organic frameworks (MOFs) [10,11], and nanostructured systems. These advances have enabled a broad spectrum of biomedical applications, including magnetic resonance imaging (MRI) and luminescent and near-infrared (NIR-II) optical imaging, as well as multimodal theranostics, drug delivery, and enzyme-mimetic catalysis [7,12]. In parallel, growing evidence for biologically essential roles of lanthanides, most notably their function as cofactors in methylotrophic bacteria via highly selective binding proteins such as lanmodulin (LanM) [13], has reshaped long-standing assumptions regarding the biological relevance of f-elements [14].

The above-mentioned advances highlight how lanthanide ions occupy a unique intersection between coordination chemistry, molecular recognition, and biomedical function. Their ability to couple hard-acid coordination with rich photophysical and magnetic behavior enables interactions with nucleic acids, peptides, and proteins, as we explored in this work, that are fundamentally distinct from those of transition metals or alkaline-earth ions. At the same time, the emergence of lanthanide-dependent biological systems, the development of highly stable synthetic chelates, and the rise in lanthanide-based nanostructures have expanded the conceptual boundaries of f-element chemistry in living environments. Despite the growing number of studies on lanthanide complexes, a comprehensive and mechanistically oriented synthesis of their biological interactions and biomedical applications is still lacking. This review aims to fill that gap by critically examining recent advances, with particular attention to their molecular activity, emerging therapeutic roles, and translational relevance. Therefore, this work aims to provide an integrated and mechanistically oriented overview of lanthanide complexes in biology and biomedicine, with particular emphasis on the relationship between coordination chemistry and biological function. We first outline the fundamental coordination principles governing Ln^3+^ behavior in aqueous and biological environments, then examine their interactions with key biomolecules, including DNA, peptides, and proteins. Subsequent sections highlight recent advances in diagnostic imaging, bimodal and multimodal systems, and therapeutic and drug-delivery applications, while also addressing toxicity, biodistribution, and translational challenges. By correlating coordination structure, physicochemical properties, and biological outcomes, this review seeks to identify the main trends shaping contemporary lanthanide-based bioinorganic research and to delineate future directions for the rational design of lanthanide-enabled biomedical tools.

### Methodology

The literature discussed in this review was identified through searches conducted in Google Scholar, Scopus, Web of Science, and PubMed. Search terms included “lanthanide complexes,” “lanthanide coordination,” “lanthanide biomolecular interactions,” “lanthanide peptides,” “lanthanide proteins,” “lanthanide DNA,” “lanthanide imaging,” and “lanthanide biomedicine,” used individually and in combination. The search covered publications from 1963 to 2025, with particular emphasis on studies published between 2020 and 2026, which represented more than 60% of all included references and reflect the rapid expansion of the field in recent years. Studies were included when they were peer-reviewed, written in English, and provided experimental or theoretical insights into lanthanide coordination chemistry, biomolecular interactions, and imaging applications, therapeutic systems, supramolecular assemblies, or biologically relevant mechanisms. Reviews were considered when they offered essential conceptual or historical context. Exclusion criteria involved retracted articles, duplicate records across databases, non-English publications, non-peer-reviewed material, conference abstracts, and studies lacking relevance to lanthanide chemistry or its biological and biomedical applications. Selection criteria included general relevance to the topic, scientific soundness, and contribution to the mechanistic or biomedical aspects discussed in the review. In total, 122 references were included in this work after the selection process.

## 2. Coordination Chemistry of Ln^3+^ in Biological Context

The lanthanides (or lanthanoids) Ln constitute a coherent group of fifteen metallic elements, from lanthanum (La, Z = 57) to lutetium (Lu, Z = 71). Their general electronic configuration is [Xe]4f^n^5d^1^6s^2^, where *n* = 0–14, and in the trivalent oxidation state—the most stable and prevalent in solution—they adopt [Xe]4f^n^ [6]. Other oxidation states are also accessible under specific conditions. The +2 state is found for Eu, Sm, and Yb, stabilized by the half-filled 4f^7^ configuration in Eu^2+^ ([Xe]4f^7^), while the +4 state is observed in Ce^4+^ and Tb^4+^, due to the energetic preference for an empty 4f shell ([Xe]4f^0^). The redox behavior of these species provides a handle for tuning electronic and photophysical properties in catalysis and sensing applications [6,15].

**Figure 1:**
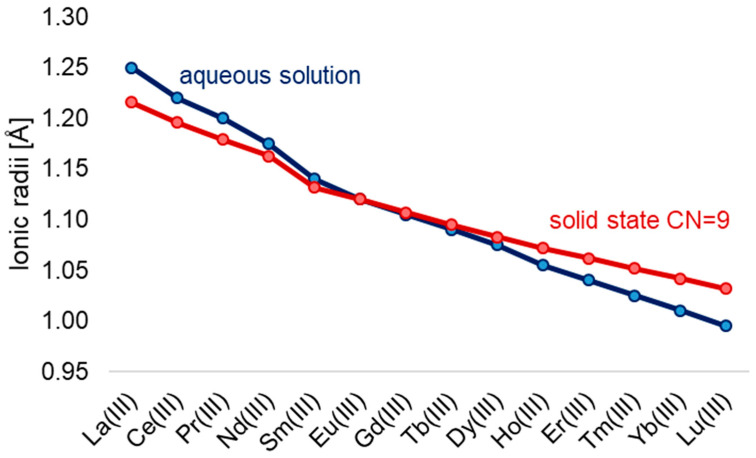
Ionic radii of Ln^3+^ in aqueous solutions [9] and in solid-state CN = 9 [17].

A defining feature of the series is the lanthanide contraction (Figure 1), a systematic decrease in ionic radii from La^3+^ (1.16 Å) to Lu^3+^ (0.97 Å) in aqueous solution [9]. This effect influences not only coordination number and geometry but also ligand-binding strength, basicity, and solubility of the resulting compounds [16].

The reduction in radius across the series also increases the Lewis acidity of the ions, enhancing their affinity for electron-donating ligands. Lanthanide ions have a comparable size to calcium (Ca^2+^, ~1.0 Å) [18], explaining their biological mimicry in Ca^2+^-binding sites [19], though their higher charge density and ionic character typically yield higher coordination numbers and stronger ligand binding [8].

**Figure 2:**
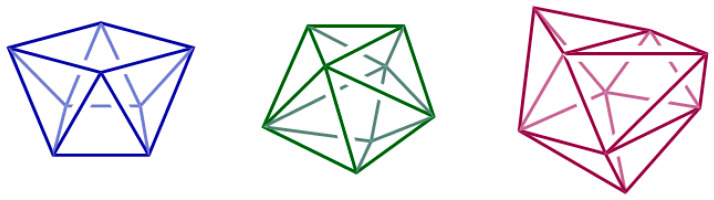
Typical coordination polyhedra of Ln^3+^ complexes: square antiprism (CN 8), dodecahedron (CN 8) and tricapped trigonal prism (CN 9).

Lanthanide ions are hard Lewis acids according to Pearson’s HSAB (Hard-Soft Acid Base) theory [20], displaying a marked preference for hard, negatively charged donors, particularly oxygen-containing ligands such as carboxylates, phosphates, phenolates, and carbonyl oxygens [16]. Nitrogen donors from amides or amines can also participate, but generally with weaker affinity. Bonding in lanthanide complexes is predominantly ionic, with little directionality or covalent character due to the inner nature of the 4f orbitals [21]. As a result, coordination geometries are dictated primarily by electrostatic and steric factors. Typical coordination numbers (CN) range between 8 and 11 [17,22,23,24], and coordination polyhedra may include square antiprismatic, dodecahedral, or tricapped trigonal prismatic arrangements [18] (Figure 2).

In aqueous solution, the larger lanthanides (La^3+^-Nd^3+^) are hydrated by nine water molecules, forming tricapped trigonal prismatic [Ln(H_2_O)_9_]^3+^ species. Smaller ions (Gd^3+^-Lu^3+^) tend to adopt eight-coordinate, square-antiprismatic [Ln(H_2_O)_8_]^3+^ structures, while mid-series ions (Pm^3+^-Eu^3+^) exhibit an equilibrium between both species [17]. The hydration number significantly affects luminescence lifetime, relaxation efficiency in MRI contrast agents, and kinetic stability of chelates. Lanthanide contraction not only compresses ionic size but also modifies the coordination geometry preferences, influencing both thermodynamic and kinetic parameters of ligand exchange reactions. For example, smaller, heavier lanthanides tend to form more rigid complexes with reduced water-exchange rates, a feature crucial for optimizing MRI contrast efficiency [25].

In neutral or slightly basic media, Ln^3+^ ions readily undergo hydrolysis, forming [Ln(OH)]^2+^, [Ln_2_(OH)_2_]^4+^, and higher-order species. The tendency increases along the series due to enhanced charge density, which promotes nucleophilic attack by water. Hydrolysis and polymerization can lead to insoluble hydroxides, complicating biological or analytical studies. Complexation with multidentate ligands suppresses hydrolysis by stabilizing the metal ion in solution. The stability constant of lanthanide-ligand complexes depends primarily on ligand denticity, charge, and geometry. Chelation modulates not only stability but also pharmacokinetics. Hydrophilic polyaminocarboxylate ligands increase renal clearance, reducing toxicity [7]. The introduction of macrocyclic and heteroatom-rich ligands further stabilizes lanthanide complexes against transmetallation. Recent strategies include conjugation with peptides or biomolecules to target specific cells or tissues, improving biocompatibility and selective uptake.

The gradual increase in Lewis acidity across the series translates into stronger binding constants for the heavier lanthanides. Chelation strength follows the order La < Ce < … < Lu. Because of their hard-acid nature, Ln^3+^ ions exhibit negligible affinity toward soft donors such as thiols or phosphines. In biological systems, they thus preferentially interact with oxygen-rich environments—carboxylates of aspartate or glutamate, phosphate groups of nucleotides, and carbonyl oxygens of peptide backbones [7].

Their affinity toward oxygen donors underlies the design of highly stable chelating agents used in biomedical applications. Macrocyclic ligands such as DOTA, DO3A, and their phosphonate derivatives [26] encapsulate the metal ion, providing thermodynamic stability and kinetic inertness suitable for in vivo applications [25,27,28]. Hydrophilic polyaminocarboxylate frameworks, on the other hand, improve aqueous solubility and biodistribution, forming the basis of clinically approved gadolinium MRI agents [7].

Because of their comparable ionic radius (Figure 1) to calcium and strong preference for oxygen donors, lanthanides can bind to biological macromolecules that normally coordinate Ca^2+^, including enzymes, cell-membrane channels, and nucleic acids. This property has been exploited in bioimaging and biosensing, where Ln^3+^ ions act as paramagnetic or luminescent probes [16]. Hydration and coordination environment also modulate photophysical and relaxometric behavior. For luminescent lanthanides such as Eu^3+^ and Tb^3+^, minimizing inner-sphere water molecules enhances quantum yield by reducing non-radiative deactivation. Conversely, for MRI-active Gd^3+^, maintaining at least one coordinated water molecule is essential for efficient relaxation of nearby protons.

## 3. Interactions with Biomolecules

### 3.1. DNA and Nucleic Acids

Among the various biomolecular partners of lanthanide ions, nucleic acids stand out as particularly well-studied systems, offering rich insights into coordination modes and biological consequences [29]. Binding occurs mainly through phosphate oxygen atoms or nucleobase carbonyls, via electrostatic or coordinative interactions.

#### 3.1.1. Direct Ln^3+^-DNA Interactions

The study of Ru et al. [30] demonstrates that trivalent lanthanide ions promote DNA compaction through a combination of electrostatic screening, ion-ion correlation, and specific localized interactions that extend beyond classical Manning–Oosawa counterion condensation theory [31,32]. While electrostatic correlation effects are substantial for trivalent ions, the observed ion-dependent behavior cannot be explained solely by valence-based models or by treating counterions as point charges. Instead, the data reveal clear ionic specificity: Ho^3+^ induces the strongest DNA compaction, followed by Pr^3+^, Tb^3+^, La^3+^, and Ce^3+^, indicating contributions from factors such as lanthanide contraction and differences in effective ion size [9], f-electron configuration effects [33], and hydration structure and dehydration energetics during binding [34]. Specific binding to DNA bases, previously documented for transition-metal and rare-earth ions [35,36,37,38], likely also contributes to the distinct affinities observed. Chelation experiments with EDTA further support this hierarchy, as the restoration of DNA length follows the same order of complex stability, with Ho^3+^-DNA being the most resistant to chelation. Remarkably, lanthanide-induced DNA compaction is both concentration- and force-dependent, with ionic specificity constituting a significant determinant of the binding and decompaction dynamics. Owing to their high charge density and strong affinity for oxygen-rich phosphate backbones, lanthanide salts promote ratiometric DNA aggregation across diverse constructs, including single-stranded (ss) DNA, four-way junctions (4WJs) [39], and quadruple helical DNA, also known as G-quadruplex (G4) DNA [40]. The formation of these condensates has been confirmed by complementary biophysical and imaging techniques, dynamic light scattering, electrophoretic mobility shift assays, and field-emission Scanning Electron Microscopy, revealing characteristic beads-on-a-string morphologies. The process is programmable: strategically placed Fluorescence resonance energy transfer (FRET) dyes on oligonucleotides generate distinct fluorescence signatures upon lanthanide binding, allowing aggregation to be monitored in real time and enabling extraction of lanthanide–DNA and lanthanide–ligand binding constants. Importantly, aggregation is reversible; addition of specific lanthanide-binding ligands can disassemble the condensates, offering a tunable handle over supramolecular behavior. A notable feature of this chemistry is the ability of lanthanides to rapidly induce and stabilize G4 structures [41,42,43], much faster than traditional Na^+^/K^+^-driven folding pathways, and to generate intermolecular G4-lanthanide assemblies that resist enzymatic degradation, including DNase I cleavage. These condensates also exhibit selective interactions with porphyrinic and amyloid-sensing dyes [44], reflecting altered supramolecular organization. Together, these findings highlight the unique capacity of lanthanides to template dynamic, reversible ODN assemblies and position them as powerful tools in DNA nanotechnology, with potential applications in biosensing, targeted delivery using aptamer-integrated scaffolds, and the design of nucleic-acid-based artificial organelles [45].

#### 3.1.2. Lanthanide Complexes and G-Quadruplexes

**Figure 3:**
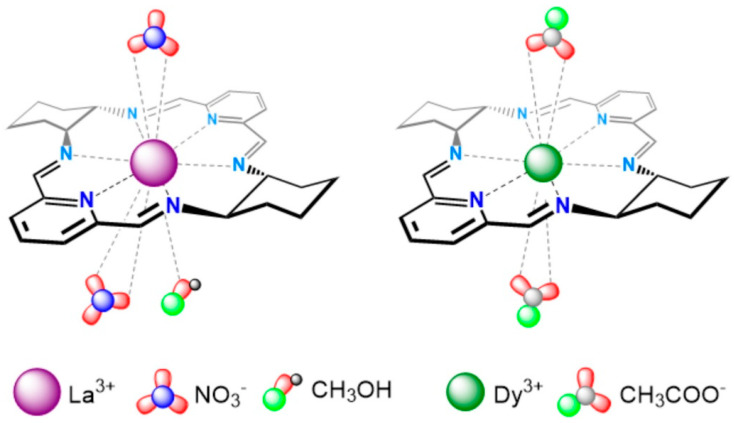
Schematic representation of macrocycles [LaL(CH_3_OH)(NO_3_)_2_]^+^ and [DyL(CH_3_COO)_2_]^+^. Reproduced from [22] © Ewert, E.; Marcinkowski, D.; Pospieszna-Markiewicz, I.; Palumbo, R.; Hnatejko, Z.; Kubicki, M.; Gorczyński, A.; Wieczorek-Szweda, E.; Patroniak, V.; Roviello, G.N, Fik-Jaskółka, M. 2025. Available under a Creative Commons CC BY license (https://creativecommons.org/licenses/by/4.0/; accessed 30 December 2025) from https://doi.org/10.1016/j.ijbiomac.2025.148269 (accessed 30 December 2025).

G4 DNA structures are important therapeutic targets in oncology. It was recently reported that macrocyclic La^3+^ (Figure 3, left) and Dy^3+^ (Figure 3, right) complexes stabilize hybrid-type and parallel G4 structures derived from telomeric and oncogenic sequences such as Tel26 and Pu22, while inducing minimal changes in duplex DNA. NMR titrations and docking studies show that coordination of the metal center may involve O6 atoms of guanine residues, stabilizing G-tetrads and sometimes inducing G4 folding from random coil states [22,23].

**Figure 4:**
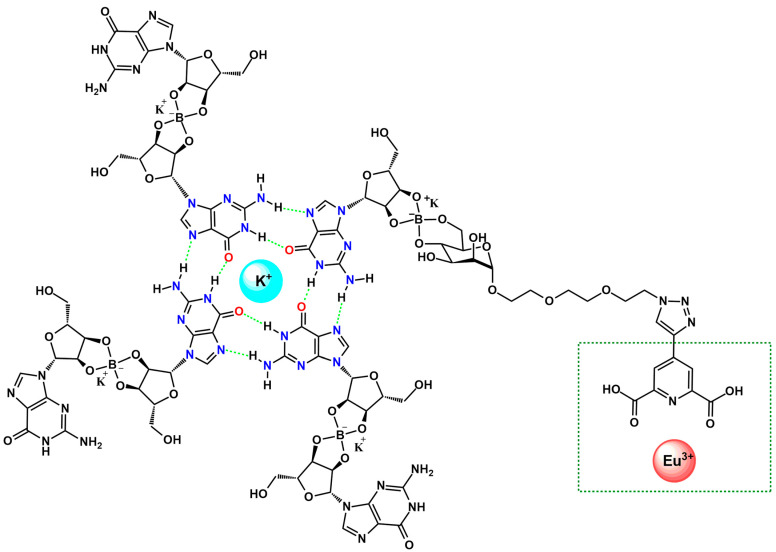
Schematic representation of a K^+^-stabilized G4 functionalized with a picolinic ligand, incorporated into a guanosine-based quadruplex architecture via its mannose moiety. The Eu^3+^ coordination site is highlighted in green [46].

Another study [46] demonstrates that incorporation of picolinic ligand into guanosine-based quadruplex architectures via its mannose moiety enables the construction of higher-order supramolecular assemblies in the presence of Eu^3+^ ions (Figure 4). Coordination of Eu^3+^ through the 2,5-dipicolinic acid unit of picolinic ligand drives the formation of robust, red-emissive hydrogels whose morphology, luminescence, and rheological behavior reflect densely interconnected networks containing crystalline guanosine dihydrate domains. Two of these materials (G4-1 and G4-2) exhibited notable stability and partial self-healing, indicating the structural reinforcement afforded by Eu^3+^-nucleoside interactions. Spectroscopic analyses, including CD (circular dichroism) and CPL (circularly polarized fluorescence) for G4-2, confirmed the emergence of a Eu^3+^-centered CPL response, with glum values lower than those observed for solution-phase Eu^3+^/1 assemblies, an effect attributed to guanosine-induced attenuation and to scattering and depolarization within the gel matrix. The inversion of the CPL signal was consistent with the chiral arrangement of guanosine quadruplex frameworks, and the overall CPL magnitude remained comparable to that of chiroptical organic emitters and quantum dots. Although the precise supramolecular organization warrants further investigation, such Eu^3+^-stabilized G-based gels represent compelling model environments for probing biomolecular interactions, particularly in light of the recently emerging biological activity of lanthanide ions [47].

The Eu^3+^–induced assembly also demonstrates responsiveness to competing cations and external stimuli, indicating potential for switchable materials and smart supramolecular systems. Overall, the results underline that Eu^3+^-guanosine binding can also be harnessed to construct functional soft materials with promising utility in bioassays, molecular recognition, and responsive biopolymer-based technologies [46].

#### 3.1.3. DNA Cleavage and Therapeutic Relevance

Ln^3+^ complexes have also been reported to promote DNA cleavage under physiological conditions, suggesting potential gene-editing or anticancer applications as reported by Jastrząb et al. in 2019 in a thorough review report [48]; therefore, herein, we will focus only on advancements from 2020 and later.

**Figure 5:**
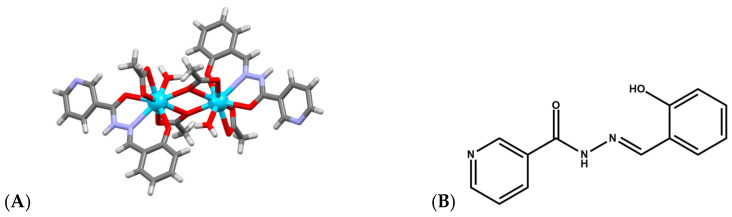
(**A**) Crystal structure of La_2_(L)_2_(μ_3_-OAc)(H_2_O)_2_]·2H_2_O (CCDC: 1857056); (**B**) Structure of ligand HL N′-(2-hydroxybenzylidene)nicotinohydrazide [52]. Color code: La, azure; N, purple; C, gray; O, red.

**Figure 6:**
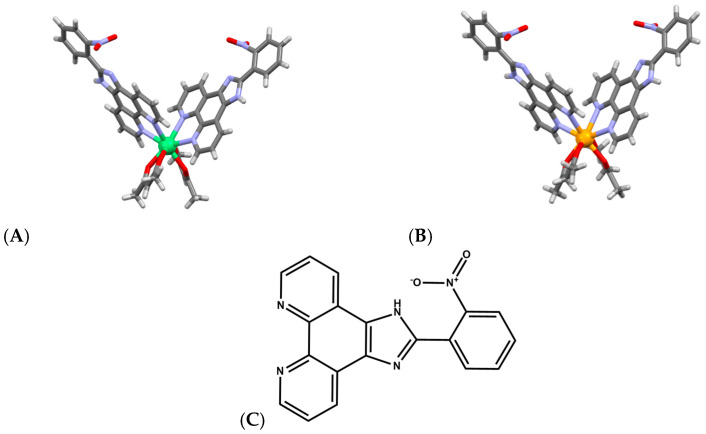
Crystal structures of (**A**) [Er(acac)_2_(o-NPIP)_2_](CH_3_CH_2_OH)_2_ (CCDC: 927657) and (**B**) [Yb(acac)_2_(o-NPIP)_2_](NO_3_)_2_ (CCDC: 2039822); (**C**) Structure of ligand 2-(2-nitrophenyl)imidazo[4,5-f]1,10-phenanthroline(o-NPIP) [53]. Color code: Er, green; Yb, orange; N, purple; C, gray; O, red.

Lanthanide complexes continue to attract significant attention as artificial nucleases because many Ln^3+^ ions exhibit strong Lewis acidity, high coordination flexibility, and tunable ligand-exchange kinetics, enabling them to promote hydrolysis or oxidative scission of DNA with implications for anticancer, antimicrobial, and diagnostic applications. A diverse set of coordination architectures, including discrete dinuclear complexes, Schiff-base assemblies, polyoxometalate (POM) [49,50,51] clusters, and MOFs, demonstrates that the DNA-cleavage behavior of lanthanides is highly sensitive to the identity of the metal center and to the structural environment created by the ligand framework. For example, La^3+^, Eu^3+^, and Dy^3+^ dinuclear hydrazide complexes of a general formula Ln_2_(L)_2_(μ_3_-OAc)(H_2_O)_2_]·2H_2_O (Figure 5A), where HL is N′-(2-hydroxybenzylidene)nicotinohydrazide (Figure 5B) were shown to bind calf-thymus DNA through a moderate intercalative mode and promote DNA cleavage in the presence of H_2_O_2_ [52]. Their cytotoxicity profiles correlate with their ability to induce DNA damage and suppress DNA synthesis, particularly in A549 cancer cells, where they trigger apoptosis more selectively than cisplatin, suggesting that controlled ROS (Reactive Oxygen Species) mediated nuclease activity from specific lanthanides may offer a therapeutic window. Analogously, [Er(acac)_2_(o-NPIP)_2_](CH_3_CH_2_OH)_2_ and [Yb(acac)_2_(o-NPIP)_2_](NO_3_)_2_ complexes bearing acetylacetonate (acac) and nitrophenyl-imidazophenanthroline (2-(2-nitrophenyl)imidazo [4,5-f]1,10-phenanthroline(o-NPIP)) ligands (Figure 6) were able to intercalate into the DNA helix and exhibit oxidative cleavage, highlighting that both heavier and middle-series lanthanides possess effective nuclease-like reactivity when combined with π-extended aromatic ligands capable of stabilizing intercalation and facilitating redox activation [53].

**Figure 7:**
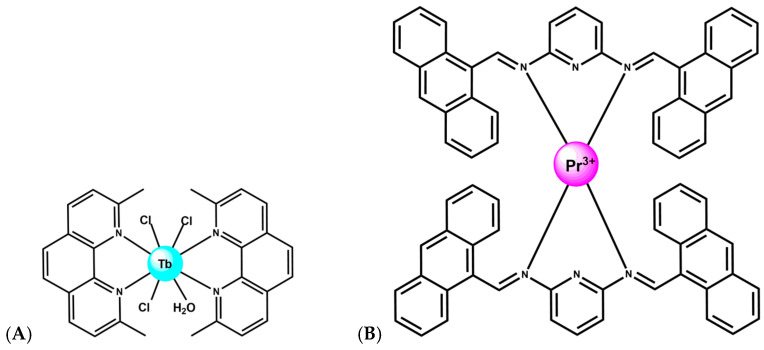
(**A**) Structure of complex [Tb(Me_2_Phen)_2_Cl_3_(OH_2_)] [54,55]. (**B**) Structure of Schiff-Pr complex with N^2^,N^6^-bis (anthracen-9-ylmethylene) pyridine-2,6-diamine ligand [56].

Tb^3+^ complexes, in particular, have emerged as multifunctional agents capable of DNA binding, cleavage, and therapeutic action. A Tb–phenanthroline complex [Tb(Me_2_Phen)_2_Cl_3_(OH_2_)] (Figure 7A), where Me_2_Phen is 2,9-dimethyl phenantroline, demonstrated strong affinity for both DNA and BSA, primarily via groove binding, and exhibited direct DNA cleavage as well as antimicrobial and anticancer properties [54,55]. Notably, encapsulation of this Tb^3+^ complex into lipid- and starch-based nanocarriers enhanced anticancer activity, highlighting the translational relevance of lanthanide-mediated DNA scission when combined with drug-delivery platforms. Schiff-Pr complex with N^2^,N^6^-bis (anthracen-9-ylmethylene) pyridine-2,6-diamine ligand likewise shows promising anticancer behavior, with activity attributed to strong metal-ion interactions with cellular components, ultimately leading to DNA fragmentation and apoptosis (Figure 7B) [56]. These findings illustrate that lanthanides with variable f-orbital occupancy, Tb^3+^ (4f^8^), Dy^3+^ (4f^9^), Pr^3+^ (4f^2^), participate in diverse cleavage mechanisms ranging from oxidative chemistry to direct coordination-driven activation of phosphodiester bonds, depending on ligand environment and cellular context.

**Figure 8:**
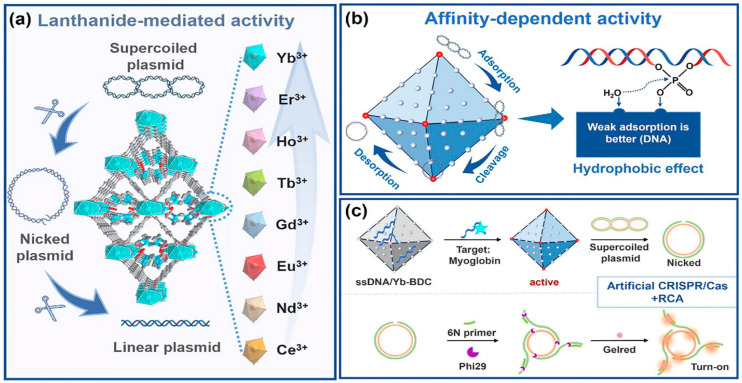
Schematic illustration of lanthanide MOF nuclease nanozymes. (**a**) A family of Ln-BDCs for cleaving DNA. (**b**) Affinity-driven mechanism: hydrophobic DNA binding governs Ln-MOF nuclease-like activity. (**c**) Yb-BDC-triggered rolling circle amplification for biosensing. Reproduced from [57]. © Gan, Z.; Yu, L.; Liu, Y.; Feng, Y.; Tong, J.; Xiao, Y. 2025. Available under a Creative Commons CC BY license (https://creativecommons.org/licenses/by/4.0/; accessed 30 December 2025) from https://doi.org/10.1002/agt2.70180 (accessed 30 December 2025).

Beyond discrete molecular complexes, Ln-containing extended frameworks display a fundamentally distinct and increasingly important mode of nuclease activity. Lanthanide MOFs (Ln-MOFs), including nanosheets and bulk crystalline materials, function as heterogeneous artificial nucleases with strong catalytic potential. For example, Yb-BDC MOF acts as a highly efficient hydrolytic nuclease mimic capable of cleaving plasmid DNA with a half-life of ≈30 min (Figure 8) [57]. Interestingly, its high activity does not stem from strong binding of the DNA substrate but rather from weak, rapidly reversible interactions that promote substrate turnover—a mechanistic distinction from traditional Lewis-acid-based phosphodiester hydrolysis probed by BNPP (bis(p-nitrophenyl) phosphate). This mechanistic insight is therapeutically relevant: rapid, catalytic, and regenerable DNA cleavage suggests potential for MOF-based chemotherapeutics or for programmable, enzyme-free nucleic-acid processing in biotechnology. Indeed, Yb-BDC successfully substituted for natural restriction enzymes to generate circular templates for rolling-circle amplification (RCA), enabling construction of a CRISPR/Cas-inspired biosensing platform.

**Figure 9:**
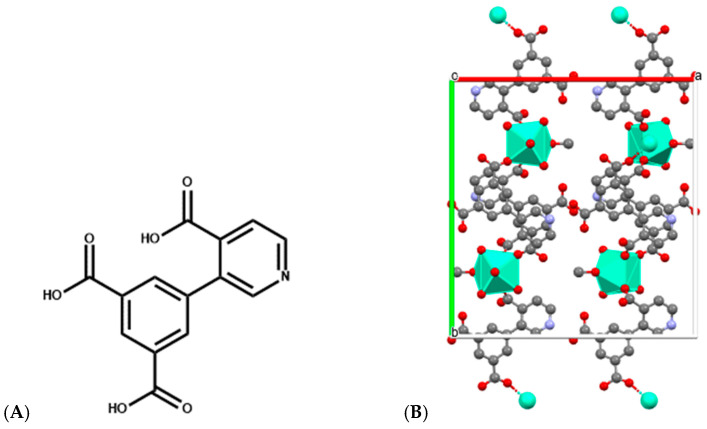
(**A**) Structure of 3-(3,5-dicarboxyphenyl) isonicotinic acid used for construction of Ln-DPIA MOFs; (**B**) Single crystal structure of Dy-DPIA MOF—two-dimensional structural diagram along the c-axis crystallographic direction (CCDC: 2341678) [58]. Color code: Dy, green; N, purple; C, gray; O, red; a, b and c are crystallographic axes.

Similarly, Eu-, Sm-, and Dy-DPIA MOF nanosheets, in which DPIA is 3-(3,5-dicarboxyphenyl) isonicotinic acid (Figure 9), are implicated in the metal coordination, exhibit both cytotoxic activity toward BxPC-3 pancreatic cancer cells and the ability to intercalate and cleave DNA, with Eu-DPIA showing the strongest DNA interaction and highest IC_50_ potency [58]. These nanosheets exemplify how structural tunability in MOFs can optimize DNA binding modes and cleavage rates for therapeutic or diagnostic applications.

**Figure 10:**
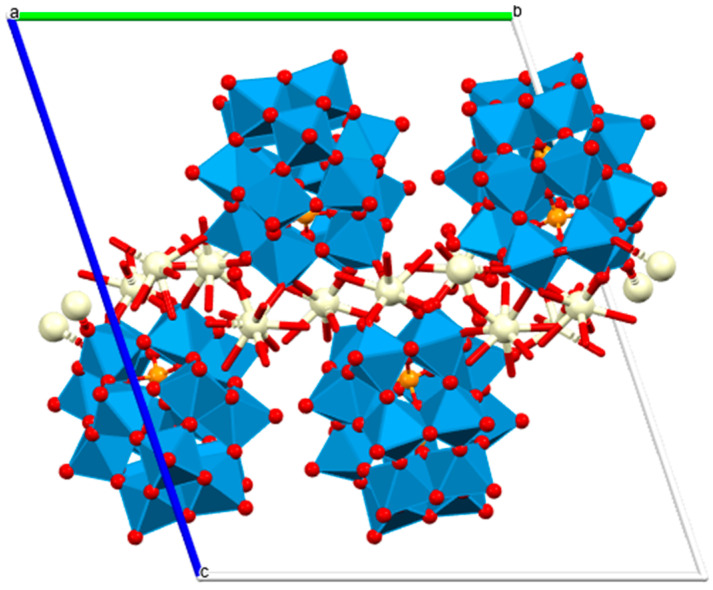
Polyhedral/ball and stick view of [[(P_2_W_17_O_61_)Ce(H_2_O)_4_]_2_[Ce_0.5_(H_2_O)_2.5_][Ln_2_(H_2_O)_14_]]^6.5−^—two-dimensional structural diagram along the a-axis crystallographic direction (CCDC: 2240783, unpublished structure) [59]. Color code: WO_6_ octahedra, blue; Ce, beige; P, orange; O, red; a, b and c are crystallographic axes.

Lanthanide polyoxometalates provide another structurally defined class of hydrolytic catalysts capable of promoting phosphodiester cleavage. Ce^3+^, Pr^3+^, and Nd^3+^ monolacunary Dawson-type POM frameworks of a general formula [[(P_2_W_17_O61)Ce(H_2_O)_4_]_2_[Ce_0.5_(H_2_O)_2.5_][Ln_2_(H_2_O)_14_]]^6.5−^ (Figure 10) catalyze BNPP hydrolysis with pseudo-first-order kinetics on the order of 10^−6^ s^−1^, forming inorganic phosphate and p-nitrophenolate as final products [59]. While BNPP is a model substrate, these results imply that f-element-bridged POMs provide rigid, multinuclear active sites that may feasibly activate more biologically relevant phosphodiesters, an idea supported by growing evidence of POM–DNA interactions in related systems. The redox flexibility of Ce^3+^/Ce^4+^, along with the Lewis acidity and high coordination numbers across the early lanthanides, suggests that these frameworks could be further engineered for DNA scission mechanisms relevant to therapeutic nucleases or controlled gene regulation.

**Figure 11:**
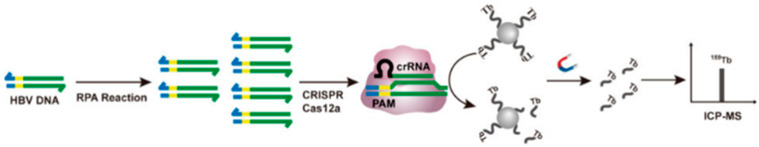
Schematic diagram of RPA coupled with CRISPR/Cas12a trans-cleavage of Tb-ssDNA-MBs for HBV DNA detection. Tb-tagged CRISPR/Cas12a biosensing platform. Reprinted with permission from Zhao, C.; Du, L.; Hu, J.; Hou, X. *Anal. Chem.* **2024**, 96, 15059–15065 [60]. © 2024 American Chemical Society.

A final dimension of lanthanide-associated DNA cleavage lies in hybrid bioanalytical-therapeutic platforms, where DNA cleavage enables detection rather than cytotoxicity. A Tb-tagged CRISPR/Cas12a biosensing platform utilizes Tb-ssDNA strands attached to magnetic beads as sacrificial cleavage targets; cleavage releases Tb^3+^ ions that are quantifiable by ICP-MS, allowing ultrasensitive viral DNA detection down to 1 copy μL^−1^ in serum (Figure 11) [60]. While not a therapeutic system, this example reveals how lanthanide-associated DNA cleavage can be repurposed into diagnostic formats with clinical impact.

Altogether, these studies show that lanthanide complexes, MOFs, and POMs offer a versatile palette of mechanisms for DNA cleavage—from oxidative and hydrolytic pathways to catalytic turnover and intercalative activation—governed by the identity of the lanthanide (La^3+^, Eu^3+^, Tb^3+^, Dy^3+^, Er^3+^, Yb^3+^, Pr^3+^, Nd^3+^) and the architecture of the surrounding ligand or framework. Their biological consequences span apoptosis induction, suppression of DNA synthesis, synergistic antimicrobial activity, and targeted anticancer effects. At the same time, the catalytic precision and modularity of lanthanide systems create opportunities for enzyme-free nucleic acid manipulation in diagnostics. This combination of therapeutic potency and technological versatility positions lanthanide-based DNA-cleaving assemblies as a rapidly advancing frontier in inorganic chemical biology and biomedical materials science.

### 3.2. Protein and Peptide Binding

Lanthanide coordination with peptides and proteins plays a central role in both fundamental bioinorganic chemistry and the development of functional probes for imaging and sensing. A comprehensive overview of this field is provided in a recent review on Ln^3+^ -binding peptides and proteins, including the study of the coordination properties and their applications [61]. Importantly, lanthanide ions bind strongly to peptide motifs enriched in Asp and Glu residues, forming inner-sphere complexes with carboxylate donors. Owing to the chemical similarity between Ln^3+^ and Ca^2+^, lanthanides can substitute for Ca^2+^ in metalloproteins, providing paramagnetic or luminescent signatures without disrupting overall protein structure. This substitution is widely exploited to obtain structural, dynamic, and functional information through paramagnetic NMR, luminescence spectroscopy, or metal-enhanced fluorescence.

**Figure 12:**
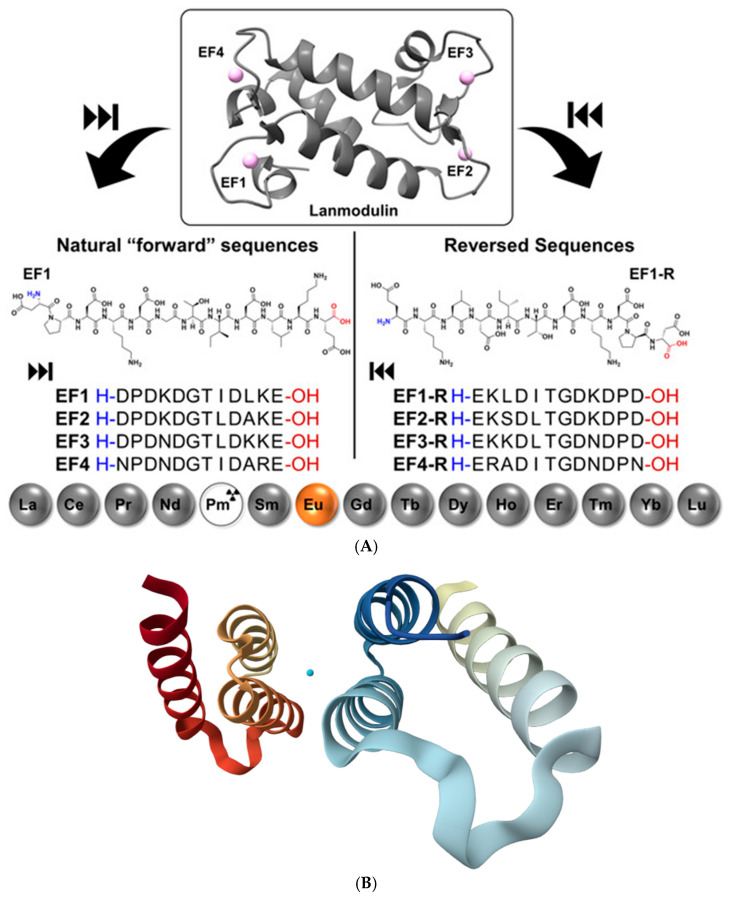
(**A**): Structure of LanM with bound neodymium (PDB 8FNS) and the sequences of the LanM-inspired peptides following the natural sequence on the right and synthesized in reverse order on the left; all peptides are shown from N to C-terminus. Reproduced from [62], © Gutenthaler-Tietze, S.M.; Daumann, L.J.; Weis, P. 2025. Available under a Creative Commons CC BY license (https://creativecommons.org/licenses/by/4.0/; accessed 30 December 2025) from https://doi.org/10.1002/ejic.202500258 (accessed 30 December 2025). (**B**): Three-dimensional structural view of the La^3+^-bound LanD protein from *Methylorubrum extorquens*, based on the freely available X-ray crystal structure PDB 9C8X (the coordinated metal ion is represented in cyan) [63] (https://www.rcsb.org/structure/9C8X; accessed 30 December 2025).

Protein and peptide binding of lanthanides has gained major attention following the discovery that Ln^3+^ ions can act as essential cofactors in biological systems. A key example is LanM, a highly selective Ln^3+^-binding protein identified in the methylotrophic bacterium *Methylobacterium extorquens*. LanM contains four EF-hand motifs (helix-loop-helix structural motifs that coordinate metal ions) typically associated with Ca^2+^ binding, yet it displays extraordinary selectivity for Ln^3+^ ions (La–Lu, Y), undergoing a pronounced conformational transition from a largely disordered to a compact, ordered structure at picomolar Ln^3+^ concentrations, while responding to Ca^2+^ only at near-millimolar levels [14]. Mutational studies revealed that conserved proline residues unique to LanM EF hands (absent in canonical Ca^2+^-binding proteins) are critical determinants of this selectivity; substitution of these residues shifts Ca^2+^ responsiveness to the micromolar range without compromising Ln^3+^ affinity. The work of Gutenthaler-Tietze et al. [62] provides a molecular framework for how biological systems discriminate scarce lanthanides from abundant alkaline-earth metals and highlights opportunities for lanthanide sensing, sequestration, and separation technologies. Building on these insights, short synthetic peptides derived from LanM metal-binding loops have been developed as selective lanthanide ligands (Figure 12A). Studies on 12-residue peptides, including sequences assembled in reverse order, demonstrate that advanced gas-phase techniques such as ion mobility spectrometry, collision-induced dissociation, and electrospray ionization reliably reproduce solution-phase binding trends and enable detailed identification of key coordinating residues. Structural insights into native lanthanide-binding proteins have been expanded by the recent 1.9 Å crystal structure of LanD from the already mentioned *Methylorubrum extorquens* in complex with La^3+^ (PDB: 9C8X, Figure 12B) [63]. This structure highlights the conserved coordination motifs and compact metal-binding pockets characteristic of lanthanide-dependent enzymes, reinforcing the mechanistic parallels between LanM, its derived peptides, and other natural Ln^3+^-binding systems. Together, these findings establish LanM-inspired peptides as minimal, tunable scaffolds for selective lanthanide recognition, with implications for bioinorganic chemistry, lanthanide recycling, and the design of lanthanide-binding tags.

**Figure 13:**
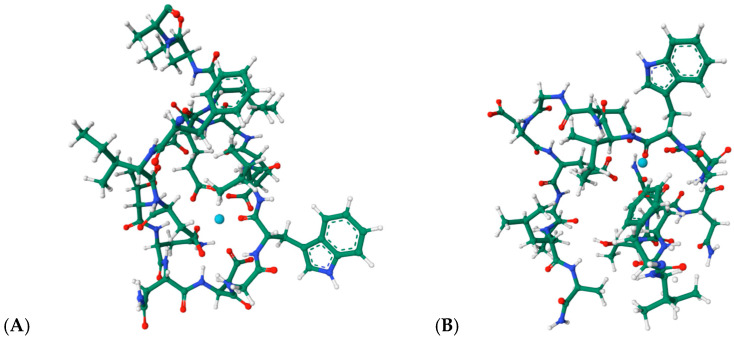
(**A**): Structural views of the lanthanide-binding tag LBT3 in complex with a La^3+^, based on the structure PDB 7CCO (https://www.rcsb.org/3d-view/7CCO/0; accessed 30 December 2025), and of (**B**) the LBT3-NH_2_:La^3+^ complex derived from PDB 9CEQ (https://www.rcsb.org/structure/9CEQ; accessed 30 December 2025). The coordinated metal ion is represented in cyan.

Engineered lanthanide-binding tags (LBTs) represent one of the most influential developments in this area. These short, 15–20-residue peptides exhibit nanomolar affinities for lanthanide ions and generate sharp f-f emission bands suitable for FRET, time-resolved luminescence, and long-lived excited-state imaging [64,65,66,67]. Their predictable coordination geometry and modularity have enabled site-specific labeling of recombinant proteins, facilitating structural mapping, conformational analysis, and tracking of protein–protein interactions in complex environments. The structural basis of lanthanide recognition by engineered tags is exemplified by the NMR structures of LBT3 bound to La^3+^ (PDB: 7CCO; 9CEQ, Figure 13A,B [68,69]). These models reveal the canonical coordination environment formed by Asp/Glu-rich loops, where inner-sphere carboxylate donors and backbone carbonyls stabilize the metal center in a compact geometry. Remarkably, these structures provide direct evidence for the predictable, modular coordination behavior that underlies the widespread use of LBTs in protein labeling, FRET, and time-resolved luminescence applications.

Beyond canonical LBTs, extensive efforts have focused on optimizing linear and cyclic peptides containing aminodiacetate or ethylenediamine triacetate donors. These scaffolds offer control over hydration number, metal coordination geometry, and peptide folding, allowing the design of responsive magnetic resonance or luminescent probes that operate through ligand rearrangement, hydration-state modulation, or metal-exchange mechanisms [61,70]. A particularly versatile class of lanthanide-binding systems is based on coiled-coil architectures. These protein-inspired ligands offer well-defined sequence–structure relationships and tunable metal-binding sites. Their main limitation is insufficient stability under physiological conditions, which has been addressed through a covalent interhelical isopeptide cross-linking strategy that markedly enhances structural integrity. Cross-linking increases thermal, kinetic, and proteolytic stability; drives the transition from an unfolded to a well-organized helical state; and improves metal-binding affinity by approximately 2.5 × 10^8^-fold relative to non-cross-linked analogues (Section 4.1.1) [71]. Overall, peptide and protein binding remains one of the most powerful approaches for harnessing the unique photophysical and magnetic properties of lanthanides, enabling precise biomolecular recognition, controlled coordination environments, and advanced imaging functionalities.

## 4. Biomedical and Therapeutic Applications

The preceding section highlighted the diverse interactions between lanthanide ions and key biomolecular targets, including nucleic acids, peptides, and proteins. These molecular-level insights provide a mechanistic foundation for understanding how lanthanide complexes exert their effects in biological systems. Building on this framework, the following sections explore how these coordination-driven interactions translate into practical biomedical applications. In particular, we examine how the unique physicochemical properties of lanthanides, combined with their biomolecular interaction-based properties, enable their use in diagnostic imaging, therapeutic delivery, and multifunctional theranostic platforms.

### 4.1. Diagnostic Imaging

Among the various applications enabled by lanthanide chemistry, diagnostic imaging remains the clearest demonstration of how coordination design translates into clinical function, a progression that reflects both foundational insights into lanthanide coordination behavior and the rapid evolution of modern imaging technologies, as we examine in greater depth below.

#### 4.1.1. Magnetic Resonance Imaging (MRI)

Lanthanide complexes, owing to their versatile magnetic properties and rich coordination chemistry, provide a broad platform for the development of molecular imaging and theranostic agents. Magnetic resonance imaging (MRI) offers excellent spatial and temporal resolution together with effectively unlimited tissue penetration. Image contrast in MRI arises from differences in longitudinal (1/T_1_) and transverse (1/T_2_) relaxation rates of water protons, and from variations in proton density across tissues. Paramagnetic compounds enhance this contrast by accelerating nuclear relaxation, predominantly through dipolar interactions with surrounding water protons. Among paramagnetic metal ions, Gd^3+^ is particularly effective because it possesses seven unpaired electrons (S = 7/2, the maximum for a metal ion) and exhibits relatively slow electron spin relaxation, resulting in exceptionally high relaxation efficiency [72,73,74]; therefore Gd^3+^ based complexes remain the foundation of MRI contrast agent design, with chelators such as DTPA and DOTA ensuring high relaxivity and reduced toxicity [7]. Beyond these classical systems, recent efforts focus on targeted, responsive, and structurally enhanced agents, complementing earlier developments summarized in [75].

**Figure 14:**
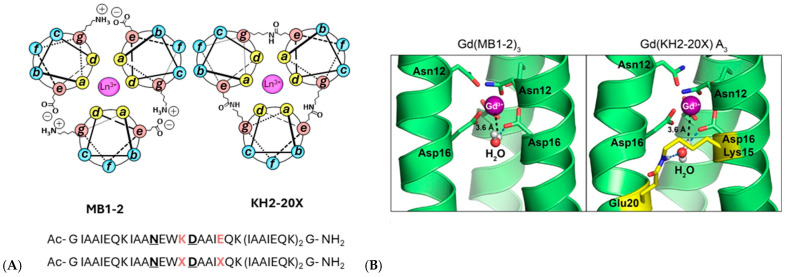
(**A**) Helical wheel representations illustrating a cross-sectional view of Ln(MB1-2)_3_ and Ln(KH2-20X), constructed according to the (abcdefg)_n_ heptad repeat. The peptide helices extend from the N-terminus (emerging from the page) to the C-terminus (projecting into the page). Interhelical salt bridges and isopeptide cross-links between Glu (e) and Lys (g) residues are indicated. Coordinated Ln^3+^ ions are shown as pink spheres. The corresponding amino acid sequences of MB1-2 (**top**) and KH2-20X (**bottom**) are included, with metal-binding residues highlighted in bold and underlined, and salt bridge (K, E) or cross-link (X) positions emphasized in bold orange. (**B**) Selected snapshots from molecular dynamics simulations of both assemblies, showing second-sphere water molecules located within the internal cavity in proximity to the Gd^3+^ ion (magenta sphere). The peptide cross-link is highlighted in yellow. Adapted from [71] © Hadley, K.A.; Ricci, M.; Hanzevacki, M.; Bernstein, H.; Jayasekera, H.S.; Leney, A.C.; Mulholland, A.J.; Carniato, F.; Botta, M.; Britton, M.M. 2025. Available under a Creative Commons CC BY license (https://creativecommons.org/licenses/by/4.0/; accessed 30 December 2025) from https://doi.org/10.1021/jacs.5c13620 (accesed 30 December 2025).

Most recently, coiled-coil peptides have emerged as tunable metal-binding scaffolds, namely MB1-2 and KH2-20X (Figure 14), and covalent interhelical isopeptide cross-linking has been shown to greatly improve their physiological stability, yielding lanthanide-bound constructs with a 2.5 × 10^8^-fold increase in metal-binding affinity, enhanced kinetic robustness, and ∼30% higher MRI efficacy through second-sphere water coordination [71].

**Figure 15:**
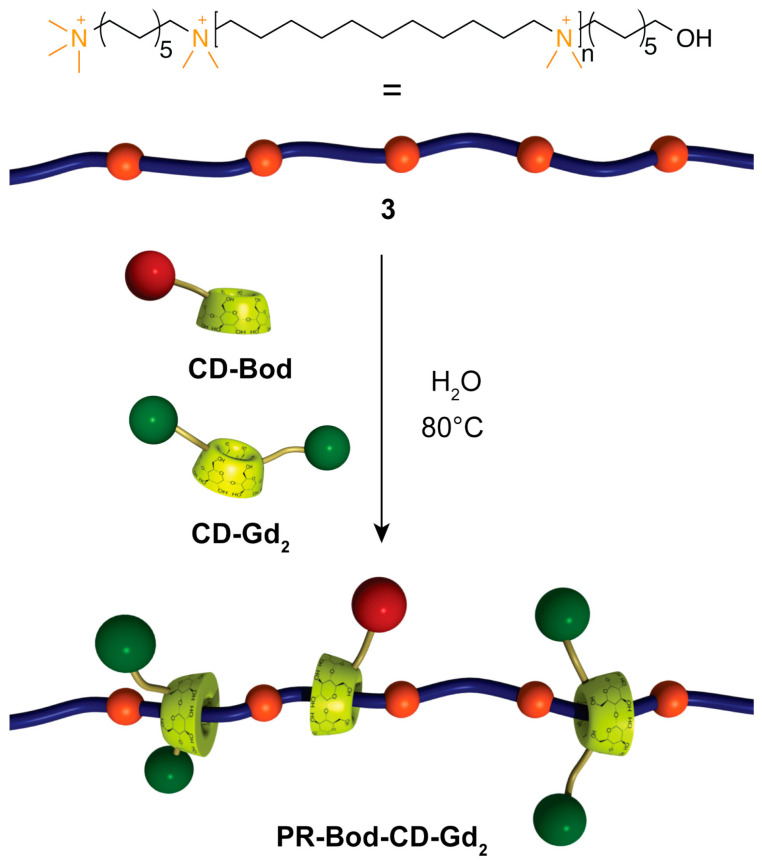
Schematic representation of the synthesis of PR-Bod-CD-Gd_2_ with partial occupation of the alkyl chains of the polymer. Reproduced from [76]. © Fredy, J.W.; Scelle, J.; Hasenknopf, B.; Tóth, É.; Vives, G.; Bonnet, C.S. 2026. Available under a Creative Commons CC BY license (https://creativecommons.org/licenses/by/4.0/; accessed 30 December 2025) from https://doi.org/10.1016/j.ica.2025.122947 (accessed 30 December 2025).

Supramolecular rotaxane and polyrotaxane architectures also offer promising relaxometric behavior: polyrotaxanes such as PR-CD-Bod-Gd_2_ (Figure 15) exhibit high rigidity, slow rotational dynamics, and relaxivities up to 19.7 mM^−1^ s^−1^ at 60 MHz and 37 °C, nearly sevenfold higher than Gd-DOTA, while detailed ^17^O NMR and NMRD analyses highlight the key roles of second-sphere contributions, steric crowding, and restricted internal motion in optimizing MRI performance [76].

**Figure 16:**
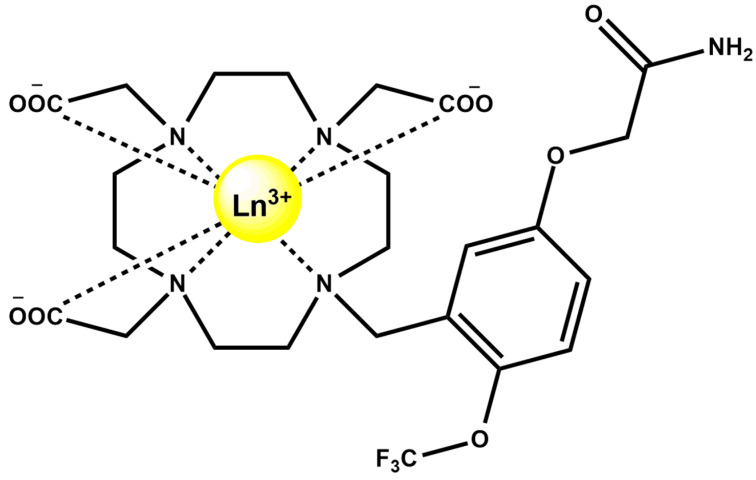
Structure of LnDO3A-based complexes, Ln^3+^ = Gd^3+^, Y^3+^, Eu^3+^, Yb^3+^, and Tb^3+^.

Complementarily, fluorinated LnDO3A-based complexes (Figure 16) incorporating Gd^3+^, Y^3+^, Eu^3+^, Yb^3+^, and Tb^3+^ provide modular multimodal probes capable of T_1_-weighted MRI, ^19^F MRI, and visible-range lanthanide luminescence, with structural consistency across the lanthanide series ensuring comparable stability and performance for cross-modality imaging [77]. Collectively, these advances illustrate how lanthanide coordination chemistry, supramolecular design, and scaffold engineering are converging to yield next-generation MRI agents with improved sensitivity, responsiveness, and multimodal functionality.

#### 4.1.2. Luminescent and NIR-II Imaging

Lanthanide-based luminescent probes have emerged as powerful tools for optical bioimaging, spanning both the visible and near-infrared spectral regions. In particular, imaging in the near-infrared II (NIR-II, 1000–1700 nm) window offers major advantages for in vivo applications, as reduced tissue scattering and autofluorescence enable deep-tissue penetration and high-resolution visualization [78,79,80]. A comprehensive and up-to-date reviews published in 2023 and 2024 systematically cover spectroscopic regulation strategies and nanoprobe engineering of NIR-II luminescent lanthanide nanocrystals for in vivo brain disease imaging, including brain injury and tumors [81,82]. Given the depth of coverage in these works, only selected recent and particularly significant advances in NIR-II lanthanide imaging are highlighted here. Alongside these developments, a substantial body of research demonstrates that Eu^3+^- and Tb^3+^-based probes emitting in the visible region remain highly effective bioimaging agents. Their characteristic red and green emissions, combined with long excited-state lifetimes and sharp emission bands, enable time-resolved detection strategies that suppress background fluorescence. Notably, dual-photosensitized Eu^3+^ and Tb^3+^ complexes with optimized antenna ligands exhibit high quantum yields in aqueous media, efficient DNA and BSA binding, and robust cellular uptake, supporting their use in cellular imaging and combined imaging–phototherapeutic applications [83,84,85].

At the molecular level, lanthanide complexes continue to demonstrate unique photophysical advantages over conventional fluorophores, including long-lived emission, sharp line-like spectra, large Stokes shifts, and the ability to participate in luminescence resonance energy transfer and upconversion processes. Recent work on a neodymium complex with nitrilotriacetic acid (NTA) ligand, [Nd(NTA)_2_·H_2_O]^3−^, illustrates how rational ligand coordination can yield efficient NIR emission suitable for bioimaging. Upon excitation at 357 nm, this complex exhibits a strong and unusually narrow emission band at 1076 nm (FWHM ≈ 10 nm), a feature not previously reported for Nd^3+^ systems. Combined experimental and theoretical analysis using Judd–Ofelt theory confirmed favorable radiative properties, supporting the applicability of such discrete lanthanide complexes as molecular NIR imaging probes [82,86].

At the nanoscale, lanthanide nanocrystals have enabled major breakthroughs in NIR-II imaging performance. A notable advance is the development of Ho^3+^-sensitized NaYF_4_-based nanocrystals excited at 1143 nm, which generate tunable emissions spanning 1000–2200 nm. Through precise core–shell engineering, these systems suppress unfavorable cross-relaxation pathways and promote efficient interfacial energy migration, resulting in multiemission outputs unattainable in conventional co-doped materials. Importantly, excitation at 1143 nm affords superior tissue penetration, particularly through highly scattering structures such as the skull, outperforming traditional 808 and 980 nm excitation [87,88]. These properties enabled six-channel NIR-II in vivo imaging of multiple organs and tumors in mice, demonstrating the feasibility of multichannel, high-contrast biological imaging using lanthanide nanocrystals [89].

Beyond steady-state luminescence, recent efforts emphasize expanding the NIR-II lanthanide toolbox toward time-domain imaging and excitation-free detection. Advances in lifetime-encoded imaging, persistent luminescence nanoparticles, and optimized excitation–emission wavelength combinations have further improved spatial resolution and penetration depth in vivo. These developments, together with parallel progress in the design of lanthanide complexes and nanoparticles, have been critically summarized in a recent article that outlines both current achievements and remaining challenges for NIR-II lanthanide probes in biological imaging [12]. Collectively, these studies point to the versatility of lanthanide luminescence across molecular and nanostructured platforms and highlight its growing impact on deep-tissue, multichannel, and high-specificity bioimaging applications.

#### 4.1.3. Bimodal and Multimodal Systems

**Figure 17:**
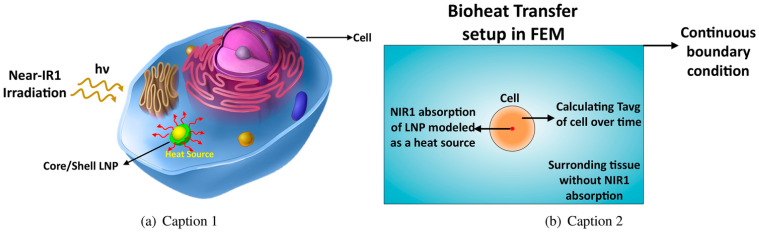
(**a**) Lanthanide-based nanoparticles (LNP) generate heat under NIR absorption inside a cell. (**b**) Modeling time-dependent bioheat transfer of NIR absorption and apoptosis event of cell. Reproduced from In-silico study of lanthanide-based nanoparticles for dual-modal photoacoustic and MRI theranostics by Aghdam, F.A.; Rostami, A. [90], under the terms of the Creative Commons Attribution–NonCommercial–NoDerivatives 4.0 International License CC BY-NC-ND 4.0 https://creativecommons.org/licenses/by-nc-nd/4.0/; accessed 30 December 2025). No changes were made.

Lanthanide-containing nanostructures serve as versatile platforms for bimodal and multimodal theranostics, particularly in the NIR region (Figure 17). Theoretical studies show that the optical and thermal behavior of lanthanide core–shell nanoparticles can be precisely tuned by adjusting core size, composition, and biocompatible coatings. DFT-derived optical constants and Mie calculations identify TiO_2_-coated particles as especially efficient NIR-I absorbers, with optimal performance at 90–110 nm and specific core–shell ratios, providing clear design rules for imaging-photothermal systems. Bioheat-transfer modeling further demonstrates tissue-dependent thermal responses, emphasizing the need to tailor nanoparticle properties to the therapeutic setting [90].

**Figure 18:**
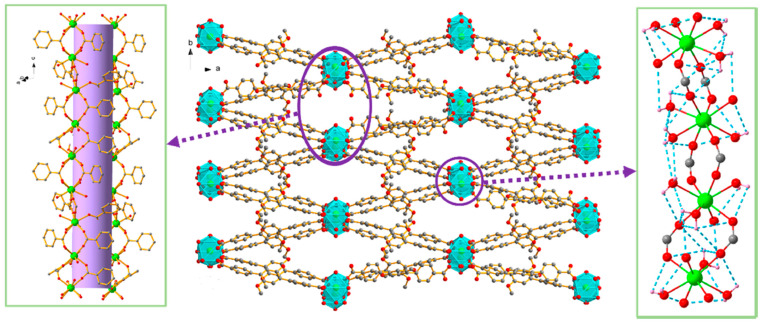
Three-dimensional structure of ([Dy(DMTP-DC)_1.5_(H_2_O)_3_]·DMF)_n_ and the 1D channel and hydrogen bonds in 4 ([Dy(DMTP-DC)_1.5_(H_2_O)_3_]·DMF)_n_. Reprinted with permission from Hu, J.-J.; Li, Y.-G.; Wen, H.-R.; Liu, S.-J.; Peng, Y.; Liu, C.-M. *Inorg. Chem.* **2022**, 61, 6819–6828 [91]). © 2022 American Chemical Society.

Lanthanide-based MOFs offer intrinsically multimodal behavior, exemplified by Eu^3+^, Gd^3+^, Tb^3+^ and Dy^3+^ frameworks built from DMTP-DC (H_2_DMTP-DC = 2′,5′-dimethoxytriphenyl-4,4″-dicarboxylic acid) ligands (Figure 18). These robust 3D materials combine lanthanide luminescence, proton conductivity, and magnetic functionality. Eu- and Tb-MOFs function as ratiometric fluorescence sensors for arginine and lysine, while all four MOFs display humidity- and temperature-dependent proton conductivity, increasing systematically from Eu^3+^ to Dy^3+^ with decreasing ionic radius. Gd-MOF exhibits a significant magnetocaloric effect, while the Dy analogue shows field-induced slow magnetic relaxation. The coexistence of optical sensing, transport properties, and magnetic response highlights their potential as multimodal theranostic platforms [91].

**Figure 19:**
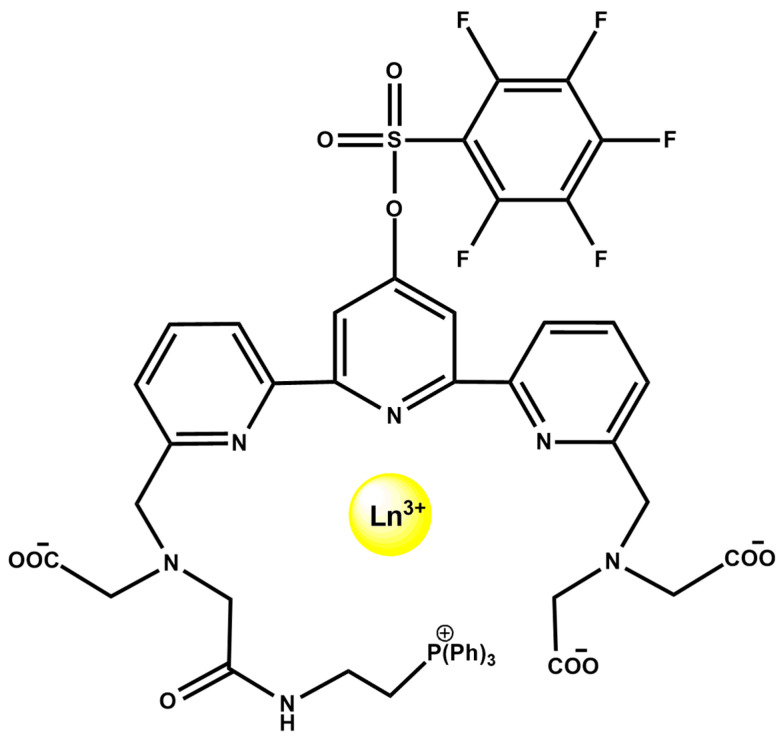
Molecular structure of Mito-PFTTA-Ln^3+^ (Ln^3+^ = Eu^3+^, Tb^3+^, or Gd^3+^).

Lanthanide coordination complexes also support integrated multimodal sensing, as illustrated by the mitochondria-targeted Mito-PFTTA-Ln^3+^ probes (Figure 19). The ligand incorporates a triphenylphosphonium targeting group and an H_2_O_2_-responsive PFBS unit, forming Eu^3+^/Tb^3+^ and Gd^3+^ complexes. The mixed Mito-PFTTA-Eu^3+^/Tb^3+^ probe functions as a ratiometric time-gated luminescence (TGL) sensor, where PFBS cleavage increases Tb^3+^ emission (540 nm) and decreases Eu^3+^ emission (610 nm), enabling mitochondrial H_2_O_2_ imaging in cells and mouse liver. In parallel, Mito-PFTTA-Gd^3+^ operates as a ^19^F MRI probe, with Gd^3+^-induced quenching of the fluorine signal reversed upon H_2_O_2_ activation, enabling ^19^F MRI in vitro and in vivo [92].

Another recent bimodal system is the liposomal BHHBSB-Eu/Gd nanoprobe, which integrates TGL with MR contrast. These β-diketone Eu^3+^/Gd^3+^ assemblies respond selectively to hypochlorous acid through oxidative decomposition that quenches Eu^3+^ luminescence and lowers Gd^3+^ relaxivity. This dual response provides sensitive detection of HClO while maintaining MRI penetration depth. The system enables visualization of exogenous and endogenous HClO in cells, zebrafish, and mouse models of liver inflammation, demonstrating the utility of lanthanide-based constructs for in vivo monitoring of inflammatory processes [93].

### 4.2. Therapeutics and Drug Delivery

Lanthanide-based systems are increasingly explored as therapeutic agents and drug-delivery platforms, owing to their rich coordination chemistry, tunable bioactivity, and compatibility with multifunctional nanostructures. Discrete lanthanide complexes have demonstrated notable anticancer potential, as exemplified by Yb-BZA and Er-BZA complexes, which interact with DNA through groove binding and exhibit strong cytotoxicity toward pancreatic cancer cells, with IC_50_ values of approximately 6 μg mL^−1^—surpassing the efficacy of oxaliplatin [94]. Similarly, dinuclear La-based coordination polymers functionalized with bioactive molecules such as Erianin enable targeted gastric cancer therapy by downregulating TMEM158, highlighting how lanthanide coordination frameworks can be engineered to modulate specific oncogenic pathways [95]. Beyond molecular complexes, lanthanide-doped nanoparticles represent a major advance in drug delivery and cancer treatment, particularly within multimodal and theranostic strategies. Surface functionalization with targeting ligands, including RGD peptides or phosphopeptides, enhances tumor selectivity and minimizes nonspecific uptake [96]. These nanoplatforms can integrate photothermal, photodynamic, and chemotherapeutic modalities, activated by external stimuli such as near-infrared light or X-ray irradiation, enabling spatiotemporally controlled therapy alongside real-time imaging feedback [97]. Protein-based carriers further extend the therapeutic versatility of lanthanides: ferritin nanocages engineered with lanthanide-binding tags can encapsulate or coordinate Tb^3+^ and Eu^3+^ ions to form luminescent, tumor-targeting bioconjugates that combine intrinsic biocompatibility with the distinctive photophysical properties of lanthanides [98]. In addition to oncology-focused applications, lanthanide complexes influence broader biological processes, including enzyme modulation; for example, Sm^3+^ and Yb^3+^ complexes catalyze phosphate ester hydrolysis under physiological conditions, mimicking nuclease activity [25]. Other hybrid Ln-flavonoid systems exhibit antibacterial effects and protein-binding affinity, highlighting the expanding therapeutic landscape of lanthanide chemistry beyond imaging and cancer treatment [99].

### 4.3. Toxicity and Biodistribution

The toxicity of lanthanides is highly speciation-dependent and cannot be discussed independently of coordination environment and formulation: genome-wide screening in the eukaryotic model *Saccharomyces cerevisiae* revealed that La^3+^ disrupted the growth of over 500 deletion strains only at higher concentrations and prolonged exposure (15 generations), a response not observed with mid- and late-series lanthanides such as Eu^3+^ and Gd^3+^, highlighting the distinct and condition-sensitive toxicity profile of early lanthanides [100,101]. Free Ln^3+^ ions disrupt Ca^2+^-dependent signaling pathways, inhibit calcium-binding proteins, and promote oxidative stress through interference with mitochondrial function, effects that translate into pronounced cytotoxicity at low micromolar concentrations in cellular systems. Importantly, chelation markedly attenuates these effects by reducing metal ion lability, limiting nonspecific protein binding, and facilitating renal or hepatobiliary clearance, thereby narrowing the toxicological window [7]. For nanostructured systems, toxicity is governed not only by the lanthanide itself but also by particle size, surface chemistry, and colloidal stability. For example, CeO_2_ nanoparticles functionalized with polyacrylic acid and RGD (Arg-Gly-Asp) peptides retained catalytic SOD- (superoxide dismutase) and catalase-like activity while significantly reducing inflammatory responses and endothelial cytotoxicity compared to unmodified analogues, demonstrating that surface engineering can decouple biological activity from toxicity [96]. Biodistribution studies further emphasize formulation-dependent safety profiles: pharmacokinetic analyses of La-based complexes revealed rapid plasma clearance and preferential accumulation in mineralized and reticuloendothelial tissues. In rats treated intravenously with 1 mg/kg/day for 5 days, lanthanum concentrations reached 16,983 ± 1160 ng/g in liver, 7817 ± 3211 ng/g in spleen, and up to ~3000 ng/g in bone (knee region), while remaining low in heart and brain (768 ± 115 ng/g and 112 ± 84 ng/g, respectively), consistent with competition between lanthanides and calcium ions for hydroxyapatite binding sites [102]. Collectively, these data indicate that ligand denticity, hydrophilicity, particle surface functionalization, and overall charge density are decisive parameters controlling lanthanide toxicity and biodistribution, and must be quantitatively optimized to achieve biologically acceptable safety profiles.

Overall, biomedicine is progressing along multiple fronts, including the emergence of supramolecular platforms such as metallogels that offer new opportunities for therapeutic applications [103], and the study of natural products implicated in pathology-related pathways [104]. Progress has also been made in elucidating mechanisms for socially relevant diseases [105], and in precision therapeutics driven by bioactive compounds, multi-omics integration, and drug-repurposing strategies [106]. Moreover, convergent strategies in neurodegeneration therapy [107], synthetic drugs for diverse therapeutic applications [108,109], together with research into antioxidant properties and biomacromolecular interactions of synthetic compounds [110,111,112,113,114,115,116,117,118,119], machine learning applications [120], and covalent inhibitors for pathogenic and cancer proteins [121] collectively highlight the diversity of current biomedical innovations. In this context, lanthanide-based biomolecular systems occupy an increasingly important position in modern biomedicine, yet several challenges remain despite the rapid progress in their development and application.

## 5. Key Challenges and Advances in Lanthanide-Based Biomedical Systems

Despite growing biomedical interest in lanthanide complexes, several fundamental questions remain unresolved. For example, a clearer mechanistic understanding is still needed as the relationship between coordination geometry, thermodynamic stability, and biological activity remains insufficiently defined. Thus, advanced computational modeling and in situ spectroscopy are needed to map these correlations [122]. Another major issue concerns controlled release and biodegradability: most lanthanide chelates are kinetically inert, limiting metabolic clearance. Hence, designing biodegradable ligands or self-immolative linkers will be key for next-generation complexes. A further challenge concerns safety and clinical translation. Gadolinium deposition disease and nanoparticle persistence highlight the need for rigorous toxicological evaluation and long-term biodistribution studies. An additional point of concern is multimodal integration. Combining magnetic, optical, and therapeutic modalities in one lanthanide platform will drive the emergence of “smart” theranostics for precision medicine [12].

In our opinion, future directions should involve hybrid systems coupling lanthanide photophysics with organic chromophores, quantum dots, proteinscaffolds, or inorganic scaffolds by forming multimodal hybrid materials (i.e., with POMs). Moreover, advances in NIR-II optical imaging and photoresponsive complexes will expand the reach of lanthanide-based biomedicine. In fact, lanthanide complexes represent a rapidly advancing frontier in bioinorganic chemistry for the last few decades, distinguished by a rare convergence of flexible coordination behavior, exceptional photophysical and magnetic properties, and emerging biological relevance. As summarized in this review, the predominantly ionic nature of Ln^3+^-ligand interactions, combined with high coordination numbers and strong affinity for oxygen donors, enables precise control over stability, hydration, and reactivity in complex biological environments. These features underpin the successful deployment of lanthanides as probes and functional agents in bioimaging, sensing, and therapy (Table 1).

The systems summarized in Table 1 illustrate the breadth of strategies by which lanthanide coordination chemistry is being translated into biological and biomedical function. A central advance lies in the precise control of lanthanide speciation and coordination environment, which directly governs stability, hydration, and signal transduction in complex biological media. Discrete chelates and peptide-based constructs demonstrate how ligand design can balance strong Ln^3+^ binding with controlled water accessibility, enabling either intense luminescence (Eu^3+^, Tb^3+^) or efficient magnetic relaxation (Gd^3+^). In particular, engineered lanthanide-binding tags and peptides provide modular platforms for protein labeling and responsive imaging, combining nanomolar metal affinity with predictable folding behavior [61,64,70].

At the nanoscale, core–shell lanthanide nanocrystals and liposomal assemblies overcome intrinsic limitations of molecular probes by enhancing brightness, circulation time, and multifunctionality. Ho^3+^-sensitized NaYF_4_-based nanocrystals enable multichannel NIR-II imaging with superior tissue penetration, while liposomal Eu/Gd systems integrate time-gated luminescence with MRI for inflammation tracking [83,93]. These examples highlight how architectural design, rather than metal identity alone, dictates imaging depth, sensitivity, and modality integration.

Supramolecular and framework-based materials further extend functionality by exploiting collective effects. Polyrotaxane Gd^3+^ assemblies achieve markedly enhanced relaxivity through slowed rotational dynamics and dominant second-sphere contributions, offering clear advantages over conventional small-molecule MRI agents [76]. Similarly, lanthanide MOFs combine optical, magnetic, and proton-conducting properties within a single crystalline platform, demonstrating that multimodal behavior can be intrinsically encoded at the structural level [91]. Beyond imaging, therapeutic relevance is increasingly evident. Discrete Yb^3+^ and Er^3+^ complexes exhibit strong DNA binding and cytotoxicity, while La^3+^ coordination polymers enable targeted anticancer mechanisms through biomolecular pathway modulation [94,95]. In parallel, nuclease-mimetic Sm^3+^ and Yb^3+^ systems and the discovery of lanmodulin highlight the emerging biological activity and selectivity of lanthanides themselves [14,25,62]. Overall, the advances summarized here reveal a unifying challenge: translating exceptional lanthanide photophysical and magnetic properties into safe, selective, and quantitatively predictable biological function. Progress across molecular, supramolecular, and nanoscale platforms indicates that this challenge is increasingly being met through rational coordination design rather than empirical optimization alone.

## 6. Conclusions

This work has examined the coordination chemistry of lanthanide ions (Section 2), their interactions with key biomolecules such as nucleic acids, peptides, and proteins (Section 3), and their translation into biomedical applications, including imaging, therapy, and drug delivery (Section 4). Collectively, the evidence gathered indicates that the unique coordination behavior and physicochemical properties of lanthanides form the basis of their biological activity and technological potential. Notably, the ability of lanthanides to interact with specific DNA structures and stabilize G-quadruplexes may offer promising avenues in anticancer and diagnostic strategies. Lanthanide coordination chemistry has expanded far beyond its classical foundations, revealing a capacity to influence biological structure and function in ways that are both subtle and profound. Across the systems discussed in this review, a common pattern emerges: lanthanide ions do not simply bind to biomolecules, but actively reorganize them. Their high charge density, flexible coordination geometries, and tunable hydration environments allow them to reshape nucleic acid topology, modulate peptide and protein folding, stabilize supramolecular assemblies, and activate catalytic pathways that are inaccessible to other metal ions. These effects are not isolated phenomena but manifestations of a broader principle that connects coordination chemistry with biological programming. In our opinion, lanthanides may become agents that encode structural transitions in DNA and G4, template the formation of nucleoside-based hydrogels, induce compaction in lanthanide-binding proteins, and modulate nuclease-like activity in molecular complexes and extended frameworks. Their coordination environment becomes a means of transmitting information, converting local chemical interactions into optical, magnetic, or catalytic outputs that report on or alter biological states. This perspective highlights the potential of lanthanides not only as probes or cofactors but as tools for actively shaping biological matter in biomolecular engineering applications. Future research should focus on elucidating structure–activity relationships in vivo, improving the selectivity and biocompatibility of lanthanide complexes, and advancing their integration into clinically viable platforms through targeted delivery systems and multimodal imaging–therapy approaches.

## Figures and Tables

**Table 1:** Representative lanthanide-based systems reported in biological and biomedical applications.

Metal Ion(s)	Formulation	Representative System/Composition	Key Results and Properties	Biological/Biomedical Application	References
Eu^3+^, Tb^3+^	Discrete complex	Lanthanide-binding tags (LBTs)	Nanomolar affinity; sharp, long-lived emission; efficient FRET and time-resolved readout	Protein labeling, FRET, time-resolved luminescence	[64]
Eu^3+^, Tb^3+^	Peptide complex	Lanthanide-binding peptides (linear/cyclic)	Controlled hydration states; tunable folding; responsive luminescence and relaxivity	Luminescent probes, MRI-responsive scaffolds	[61,70]
Nd^3+^	Discrete complex	[Nd(NTA)_2_·H_2_O]^3−^	Intense NIR emission at 1076 nm with narrow bandwidth; favorable radiative lifetime	NIR bioimaging (1076 nm emission)	[86]
Ho^3+^, Yb^3+^, Ln^3+^	Core–shell nanocrystals	NaYF_4_:Ho, Ln@NaYF_4_	Multichannel NIR-II emission; deep tissue and through-skull imaging	Multichannel NIR-II in vivo imaging	[89]
Gd^3+^	Chelated complex	DOTA/DTPA derivatives	High relaxivity; controlled water exchange; reduced toxicity	MRI contrast agents	[7]
Gd^3+^	Supramolecular assembly	Polyrotaxane PR-CD-Gd_2_	Up to seven-fold higher relaxivity than Gd–DOTA; dominant second-sphere contribution	High-relaxivity MRI contrast	[76]
Eu^3+^, Gd^3+^	Liposomal nanoparticle	BHHBSB-Eu/Gd NPs	HClO-triggered luminescence quenching and relaxivity decrease; dual readout	Bimodal TGL/MR imaging of inflammation	[93]
Eu^3+^, Tb^3+^, Gd^3+^	Molecular probe	Mito-PFTTA-Ln	Ratiometric TGL response and ^19^F MRI signal recovery upon H_2_O_2_ activation	TGL/^19^F MRI detection of H_2_O_2_	[92]
Eu^3+^, Gd^3+^, Tb^3+^, Dy^3+^	MOF	Ln-DMTP-DC frameworks	Coexisting luminescence, proton conductivity, and magnetic effects	Multimodal sensing, proton conduction, magnetism	[91]
Yb^3+^, Er^3+^	Discrete complex	Ln–BZA complexes	Groove DNA binding; IC_50_ ~ 6 μg mL^−1^; higher activity than oxaliplatin	DNA binding, anticancer activity	[94]
La^3+^	Coordination polymer	Erianin-functionalized La CP	TMEM158 downregulation; targeted gastric cancer response	Targeted gastric cancer therapy	[95]
Sm^3+^, Yb^3+^	Discrete complex	Ln–phosphate ester systems	Efficient phosphodiester hydrolysis under physiological conditions	Nuclease-mimetic catalysis	[25]
Ln^3+^ (various)	Protein	LanM	Picomolar affinity; high selectivity over Ca^2+^; EF-hand-based recognition	Selective lanthanide binding, separation	[14,62]

## Data Availability

No new data were created or analyzed in this study. Data sharing is not applicable to this article.

## Abbreviations

The following abbreviations are used in this manuscript:

4WJ	four-way junction
BNPP	bis(p-nitrophenyl) phosphate
CD	Circular Dichroism
CN	Coordination Number
CPL	Circularly Polarized Fluorescence
CRISPR	Clustered Regularly Interspaced Short Palindromic Repeats
DPIA	3-(3,5-dicarboxyphenyl) isonicotinic acid
EF-hand motif	Helix-loop-helix structural motifs that coordinate metal ions
FRET	Fluorescence resonance energy transfer
FWHM	Full-width at half maximum
G4	G-quadruplex
LanM	lanmodulin
LBT	Engineered lanthanide-binding tag
LNP	Lanthanide-based Nanoparticles
MOF	Metal–Organic Framework
MRI	Magnetic Resonance Imaging
NIR	Near-Infrared
NTA	nitrilotriacetic acid
POM	polyoxometalate
RCA	Rolling-Circle Amplification
RGD	Arg-Gly-Asp
ROS	Reactive Oxygen Species
SOD	Superoxide Dismutase
ssDNA	Single-stranded DNA
TGL	Time-Gated Luminescence

## References

[1] Bünzli J.-C.G. (2014). Lanthanide coordination chemistry: From old concepts to coordination polymers. J. Coord. Chem..

[2] Eliseeva S.V., Bünzli J.-C.G. (2010). Lanthanide luminescence for functional materials and bio-sciences. Chem. Soc. Rev..

[3] Bünzli J.-C.G. (2010). Lanthanide luminescence for biomedical analyses and imaging. Chem. Rev..

[4] Bünzli J.-C.G. (2015). On the design of highly luminescent lanthanide complexes. Coord. Chem. Rev..

[5] Bünzli J.-C.G., Eliseeva S.V. (2010). Basics of lanthanide photophysics. Lanthanide Luminescence: Photophysical, Analytical and Biological Aspects.

[6] Bunzli E.-C.G. (2006). Benefiting from the Unique Properties of Lanthanide Ions. Acc. Chem. Res..

[7] Misra S.N., Gagnani M.A., Indira Devi M., Shukla R.S. (2004). Biological and Clinical Aspects of Lanthanide Coordination Compounds. Bioinorg. Chem. Appl..

[8] Evans C.H. (2013). Biochemistry of the Lanthanides.

[9] D’Angelo P., Zitolo A., Migliorati V., Chillemi G., Duvail M., Vitorge P., Abadie S., Spezia R. (2011). Revised Ionic Radii of Lanthanoid(III) Ions in Aqueous Solution. Inorg. Chem..

[10] Marrett J.M., Effaty F., Ottenwaelder X., Friščić T. (2025). Mechanochemistry for metal–organic frameworks and covalent–organic frameworks (MOFs, COFs): Methods, materials, and mechanisms. Adv. Mater..

[11] Song Y., Li J., Chi D., Xu Z., Liu J., Chen M., Wang Z. (2025). AI-driven advances in metal–organic frameworks: From data to design and applications. Chem. Commun..

[12] Zhu X., Zhang H., Zhang F. (2023). Expanding NIR-II Lanthanide Toolboxes for Improved Biomedical Imaging and Detection. Acc. Mat. Res..

[13] Gut M., Wilhelm T., Beniston O., Ogundipe S., Kuo C.C., Nguyen K., Furst A. (2025). Lanmodulin-Decorated Microbes for Efficient Lanthanide Recovery. Adv. Mater..

[14] Cotruvo J.A., Featherston E.R., Mattocks J.A., Ho J.V., Laremore T.N. (2018). Lanmodulin: A Highly Selective Lanthanide-Binding Protein from a Lanthanide-Utilizing Bacterium. J. Am. Chem. Soc..

[15] Tsukube H., Shinoda S. (2002). Lanthanide Complexes in Molecular Recognition and Chirality Sensing of Biological Substrates. Chem. Rev..

[16] Kremer C., Torres J., Domínguez S., Mederos A. (2005). Structure and thermodynamic stability of lanthanide complexes with amino acids and peptides. Coord. Chem. Rev..

[17] Shannon R.D. (1976). Revised effective ionic radii and systematic studies of interatomic distances in halides and chalcogenides. Acta Cryst. A.

[18] Cotton F.A., Wilkinson G., Murillo C.A., Bochmann M. (1999). Advanced Inorganic Chemistry.

[19] Nikolova V., Kircheva N., Dobrev S., Angelova S., Dudev T. (2023). Lanthanides as Calcium Mimetic Species in Calcium-Signaling/Buffering Proteins: The Effect of Lanthanide Type on the Ca(^2+^)/Ln(^3+^) Competition. Int. J. Mol. Sci..

[20] Pearson R.G. (1963). Hard and Soft Acids and Bases. J. Am. Chem. Soc..

[21] Gschneidner K.A., Eyring L. (1991). Handbook on the Physics and Chemistry of Rare Earths.

[22] Ewert E., Marcinkowski D., Pospieszna-Markiewicz I., Palumbo R., Hnatejko Z., Kubicki M., Gorczyński A., Wieczorek-Szweda E., Patroniak V., Roviello G.N. (2025). La^3+^ and Dy^3+^ hexaaza macrocycles revisited: Enhanced stabilization of G-quadruplex DNA—Spectroscopic and in silico studies. Int. J. Biol. Macromol..

[23] Fik-Jaskółka M.A., Pospieszna-Markiewicz I., Roviello G.N., Kubicki M., Radecka-Paryzek W., Patroniak V. (2021). Synthesis and Spectroscopic Investigation of a Hexaaza Lanthanum(III) Macrocycle with a Hybrid-Type G4 DNA Stabilizing Effect. Inorg. Chem..

[24] Pospieszna-Markiewicz I., Fik-Jaskółka M.A., Hnatejko Z., Patroniak V., Kubicki M. (2022). Synthesis and Characterization of Lanthanide Metal Ion Complexes of New Polydentate Hydrazone Schiff Base Ligand. Molecules.

[25] Torres J., Brusoni M., Peluffo F., Kremer C., Domínguez S., Mederos A., Kremer E. (2005). Phosphodiesterolytic activity of lanthanide (III) complexes with α-amino acids. Inorg. Chim. Acta.

[26] Voráčová I., Vaněk J., Pasulka J., Střelcová Z., Lubal P., Hermann P. (2013). Dissociation kinetics study of copper (II) complexes of DO3A, DOTA and its monosubstituted derivatives. Polyhedron.

[27] Bousquet J.C., Saini S., Stark D.D., Hahn P.F., Nigam M., Wittenberg J., Ferrucci J.T. (1988). Gd-DOTA: Characterization of a new paramagnetic complex. Radiology.

[28] Magerstädt M., Gansow O.A., Brechbiel M.W., Colcher D., Baltzer L., Knop R.H., Girton M.E., Naegele M. (1986). Gd(DOTA): An alternative to Gd(DTPA) as a T1,2 relaxation agent for NMR imaging or spectroscopy. Magn. Reson. Med..

[29] He Y., Lopez A., Zhang Z., Chen D., Yang R., Liu J. (2019). Nucleotide and DNA coordinated lanthanides: From fundamentals to applications. Coord. Chem. Rev..

[30] Ru X.-M., Yang Z.-Y., Ran S.-Y. (2022). Lanthanide ions induce DNA compaction with ionic specificity. Int. J. Biol. Macromol..

[31] Manning G.S. (1978). The molecular theory of polyelectrolyte solutions with applications to the electrostatic properties of polynucleotides. Q. Rev. Biophys..

[32] Manning G.S. (1977). Limiting laws and counterion condensation in polyelectrolyte solutions. IV. The approach to the limit and the extraordinary stability of the charge fraction. Biophys. Chem..

[33] Lin W.T.D., Huang P.-J.J., Pautler R., Liu J. (2014). The group trend of lanthanides binding to DNA and DNAzymes with a complex but symmetric pattern. Chem. Commun..

[34] Rudolph W.W., Irmer G. (2020). On the Hydration of the Rare Earth Ions in Aqueous Solution. J. Sol. Chem..

[35] Bertrand H., Monchaud D., De Cian A., Guillot R., Mergny J.-L., Teulade-Fichou M.-P. (2007). The importance of metal geometry in the recognition of G-quadruplex-DNA by metal-terpyridine complexes. Org. Biomol. Chem..

[36] Arola-Arnal A., Benet-Buchholz J., Neidle S., Vilar R. (2008). Effects of Metal Coordination Geometry on Stabilization of Human Telomeric Quadruplex DNA by Square-Planar and Square-Pyramidal Metal Complexes. Inorg. Chem..

[37] Li G.-Y., Guan R.-L., Ji L.-N., Chao H. (2014). DNA condensation induced by metal complexes. Coord. Chem. Rev..

[38] Tajmir-Riahi H.A., Ahmad R., Naoui M. (1993). Interaction of calf-thymus DNA with trivalent La, Eu, and Tb ions. Metal ion binding, DNA condensation and structural features. J. Biomol. Struct. Dyn..

[39] Dettmer S.J., Stock H.M., Hannon M.J. (2025). Interactions of elongated dinuclear metallo-cylinders with DNA three-way and four-way junctions. JBIC J. Biol. Inorg. Chem..

[40] Mergny J.-L., Sen D. (2019). DNA quadruple helices in nanotechnology. Chem. Rev..

[41] Roxo C., Pasternak A. (2025). Switching off cancer–An overview of G-quadruplex and i-motif functional role in oncogene expression. Bioorg. Med. Chem. Lett..

[42] Esposito D., Locatelli A., Morigi R. (2025). Molecular Tools for Precision Targeting and Detection of G-Quadruplex Structures. Molecules.

[43] Li P., Wei Y., Liu S., Wu J., Wu Y., Yan J., Liu S., Tan X., Huang K.-J. (2025). Functional metal organic framework mediated G-quadruplex DNA nanostructures for improved self-powered smartphone-assisted dual-mode biosensing. Biosens. Bioelectron..

[44] Namboodiri V., Sarkar A., Kumbhakar M. (2025). Binding activated single molecule burst analysis highlights amyloid sensing interaction of dye SYPRO orange. Spectrochim. Acta Part A Mol. Biomol. Spectrosc..

[45] Prasad P.K., Inti A., Yadav S.P.S. (2024). Programmable Aggregation of Self-Assembled DNA Constructs. Small Methods.

[46] Kotova O., O’Reilly C., Barwich S.T., Mackenzie L.E., Lynes A.D., Savyasachi A.J., Ruether M., Pal R., Möbius M.E., Gunnlaugsson T. (2022). Lanthanide luminescence from supramolecular hydrogels consisting of bio-conjugated picolinic-acid-based guanosine quadruplexes. Chem.

[47] Cheisson T., Schelter E.J. (2019). Rare earth elements: Mendeleev’s bane, modern marvels. Science.

[48] Jastrząb R., Nowak M., Skrobańska M., Tolińska A., Zabiszak M., Gabryel M., Marciniak Ł., Kaczmarek M.T. (2019). DNA as a target for lanthanide(III) complexes influence. Coord. Chem. Rev..

[49] Chen X., Li Q., Li M., Shi M., Liang M., Chen Y., Bai F., Lan Y.Q. (2025). Frontiers in Polyoxometalate-Based Hybrids for Photothermal Catalysis. Adv. Funct. Mater..

[50] Wang Q., Wang G., Ren H., Li Z., Zhang C., Chen T., Pang H. (2025). Polyoxometalate-based materials for electrochemical energy storage and catalytic hydrogen production. Coord. Chem. Rev..

[51] Moghadasi M., Abbasi M., Mousavi M., Mirzaei M. (2025). Polyoxometalate-based materials in therapeutic and biomedical applications: Current status and perspective. Dalton Trans..

[52] Song X.-Q., Wang Z.-G., Wang Y., Huang Y.-Y., Sun Y.-X., Ouyang Y., Xie C.-Z., Xu J.-Y. (2020). Syntheses, characterization, DNA/HSA binding ability and antitumor activities of a family of isostructural binuclear lanthanide complexes containing hydrazine Schiff base. J. Biomol. Struct. Dyn..

[53] Zhao X.-F., Zhang P.-F., Guo W.-Y., Qi R.-Z., Li X., Bai J., Yang L.-H., Ouyang Y., Xu J.-y. (2022). Lanthanide (III) complexes (Ln = Er and Yb) based on polypyridyl ligand: Synthesis, crystal structure, DNA-binding activity and interaction with human serum protein in vitro. J. Mol. Struct..

[54] Aramesh-Boroujeni Z., Bordbar A.-K., Khorasani-Motlagh M., Fani N., Sattarinezhad E., Noroozifar M. (2018). Computational and experimental study on the interaction of three novel rare earth complexes containing 2,9-dimethyl-1,10-phenanthroline with human serum albumin. J. Iran. Chem. Soc..

[55] Aramesh-Boroujeni Z., Jahani S., Khorasani-Motlagh M., Kerman K., Noroozifar M. (2020). Evaluation of parent and nano-encapsulated terbium(III) complex toward its photoluminescence properties, FS-DNA, BSA binding affinity, and biological applications. J. Trace Elem. Med. Biol..

[56] Andiappan K., Sanmugam A., Deivanayagam E., Karuppasamy K., Kim H.-S., Vikraman D. (2023). Detailed investigations of rare earth (Yb, Er and Pr) based inorganic metal-ion complexes for antibacterial and anticancer applications. Inorg. Chem. Commun..

[57] Gan Z., Yu L., Liu Y., Feng Y., Tong J., Xiao Y. (2025). Engineering Lanthanide Metal-Organic Framework Nuclease Nanozymes: Unveiling Affinity-Driven DNA Hydrolysis. Aggregate.

[58] Luo X., Chong S., Li Y., Wu S., Sun Y., Zhu M., Zhang Y., Sun C. (2025). Synthesis of Eu, Sm and Dy metal-organic framework nanosheets based on pyridyl carboxylic acid and their cytotoxic mechanism in vitro. J. Mol. Struct..

[59] Yan B., Zhang M., Liu L., Song Y., Han Q., Ma P. (2025). Ln^3+^-bridged 2D Dawson-type phosphotungstates with efficient hydrolytic cleavage activity of a DNA-model phosphodiester. J. Mol. Struct..

[60] Zhao C., Du L., Hu J., Hou X. (2024). Recombinase Polymerase Amplification and Target-Triggered CRISPR/Cas12a Assay for Sensitive and Selective Hepatitis B Virus DNA Analysis Based on Lanthanide Tagging and Inductively Coupled Plasma Mass Spectrometric Detection. Anal. Chem..

[61] Falcone E., Mathieu E., Hureau C. (2025). Lanthanide(III)-binding peptides and proteins: Coordination properties and applications. Chem. Soc. Rev..

[62] Gutenthaler-Tietze S.M., Daumann L.J., Weis P. (2025). Lanthanide-Binding Lanmodulin-Based Peptides: Insights from Advanced Mass Spectrometry Techniques. Eur. J. Inorg. Chem..

[63] Larrinaga W.B., Jung J.J., Lin C.-Y., Boal A.K., Cotruvo J.A. (2024). Modulating metal-centered dimerization of a lanthanide chaperone protein for separation of light lanthanides. Proc. Natl. Acad. Sci. USA.

[64] Martin L.J., Imperiali B. (2015). The best and the brightest: Exploiting tryptophan-sensitized Tb(^3+^) luminescence to engineer lanthanide-binding tags. Methods. Mol. Biol..

[65] Franz K.J., Nitz M., Imperiali B. (2003). Lanthanide-Binding Tags as Versatile Protein Coexpression Probes. ChemBioChem.

[66] Sculimbrene B.R., Imperiali B. (2006). Lanthanide-Binding Tags as Luminescent Probes for Studying Protein Interactions. J. Am. Chem. Soc..

[67] Martin L.J., Sculimbrene B.R., Nitz M., Imperiali B. (2005). Rapid Combinatorial Screening of Peptide Libraries for the Selection of Lanthanide-Binding Tags (LBTs). QSAR Comb. Sci..

[68] Hatanaka T., Kikkawa N., Matsugami A., Hosokawa Y., Hayashi F., Ishida N. (2020). The origins of binding specificity of a lanthanide ion binding peptide. Sci. Rep..

[69] Kt S.S., Qiao B., Marmorstein J.G., Wang Y., Favaro D.C., Stebe K.J., Petersson E.J., Radhakrishnan R., de la Fuente-Nunez C., Tu R.S. (2025). The Role of Asparagine as a Gatekeeper Residue in the Selective Binding of Rare Earth Elements by Lanthanide-Binding Peptides. Chem.–A Eur. J..

[70] Cisnetti F., Gateau C., Lebrun C., Delangle P. (2009). Lanthanide(III) Complexes with Two Hexapeptides Incorporating Unnatural Chelating Amino Acids: Secondary Structure and Stability. Chem. Eur. J..

[71] Hadley K.A., Ricci M., Hanzevacki M., Bernstein H., Jayasekera H.S., Leney A.C., Mulholland A.J., Carniato F., Botta M., Britton M.M. (2025). Metallo-coiled Coil Stabilization via Chemical Cross-Linking: Implications for Gd(III)-Based MRI Contrast Agents. J. Am. Chem. Soc..

[72] Bottrill M., Kwok L., Long N.J. (2006). Lanthanides in magnetic resonance imaging. Chem. Soc. Rev..

[73] Lacerda S., Tóth É. (2017). Lanthanide Complexes in Molecular Magnetic Resonance Imaging and Theranostics. ChemMedChem.

[74] Liu S., Tegafaw T., Ho S.L., Yue H., Zhao D., Liu Y., Mulugeta E., Chen X., Lee H., Ahn D. (2025). Magnetic Resonance Imaging and X-Ray Imaging Properties of Ultrasmall Lanthanide Oxide (Ln = Eu, Gd, and Tb) Nanoparticles Synthesized via Thermal Decomposition. Molecules.

[75] Lacerda S., Djanashvili K., Bonnet C.S., Faulkner S., Gunnlaugsson T., O Maille G. (2022). Lanthanide Containing Systems for Molecular Magnetic Resonance Imaging and Therapy. Supramolecular Chemistry in Biomedical Imaging.

[76] Fredy J.W., Scelle J., Hasenknopf B., Tóth É., Vives G., Bonnet C.S. (2026). Supramolecular rotaxanes and polyrotaxanes as potential MRI contrast agents: A comprehensive 17O NMR and relaxometric study. Inorg. Chim. Acta.

[77] Jin Z., Yue P., Chen F., Chen Q., Angelovski G., Wang G. (2025). Lanthanide(III)-based complexes for potential dual 1H/19F MRI contrast agents: Synthesis, structure, relaxivity and 19F NMR spectroscopy studies. Inorg. Chem. Commun..

[78] Pu T., Liu Y., Pei Y., Peng J., Wang Z., Du M., Liu Q., Zhong F., Zhang M., Li F. (2023). NIR-II Fluorescence Imaging for the Detection and Resection of Cancerous Foci and Lymph Nodes in Early-Stage Orthotopic and Advanced-Stage Metastatic Ovarian Cancer Models. CS Appl. Mater. Interfaces.

[79] Bhuin S., Chakraborty P., Yogeeswari P., Chakravarty M. (2025). From Light to Insight: Harnessing Fluorescent Probes for Intracellular Pathway Visualization. ACS Biomater. Sci. Eng..

[80] Li C., Chen G., Zhang Y., Wu F., Wang Q. (2020). Advanced Fluorescence Imaging Technology in the Near-Infrared-II Window for Biomedical Applications. J. Am. Chem. Soc..

[81] Du Y., Ni S., Ma Q., Song X., Yang H. (2023). Engineering NIR-II luminescent lanthanide nanoprobes for imaging brain diseases in vivo. Coord. Chem. Rev..

[82] Mei M., Wu B., Wang S., Zhang F. (2024). Lanthanide-dye hybrid luminophores for advanced NIR-II bioimaging. Curr. Opin. Chem. Biol..

[83] Dasari S., Maparu A.K., Abbas Z., Kumar P., Birla H., Sivakumar S., Patra A.K. (2020). Bimetallic Europium and Terbium Complexes Containing Substituted Terpyridines and the NSAID Drug Tolfenamic Acid: Structural Differences, Luminescence Properties, and Theranostic Applications. Eur. J. Inorg. Chem..

[84] Dasari S., Singh S., Sivakumar S., Patra A.K. (2016). Dual-Sensitized Luminescent Europium(ΙΙΙ) and Terbium(ΙΙΙ) Complexes as Bioimaging and Light-Responsive Therapeutic Agents. Chem. Eur. J..

[85] Pandya S., Yu J., Parker D. (2006). Engineering emissive europium and terbium complexes for molecular imaging and sensing. Dalton Trans..

[86] Sasani Ghamsari M., Arghavan M.M. (2024). [Nd(NTA)2·H2O]3− complex with high-efficiency emission in NIR region. Heliyon.

[87] Liu X., Que I., Kong X., Zhang Y., Tu L., Chang Y., Wang T.T., Chan A., Löwik C.W.G.M., Zhang H. (2015). In vivo 808 nm image-guided photodynamic therapy based on an upconversion theranostic nanoplatform. Nanoscale.

[88] Sukul P.P., Kumar K. (2016). Near-infrared (808 and 980 nm) excited photoluminescence study in Nd-doped Y2O3 phosphor for bio-imaging. Methods Appl. Fluoresc..

[89] Wang X., Wu W., Yun B., Huang L., Chen Z.-H., Ming J., Zhai F., Zhang H., Zhang F. (2025). An Emerging Toolkit of Ho^3+^ Sensitized Lanthanide Nanocrystals with NIR-II Excitation and Emission for in Vivo Bioimaging. J. Am. Chem. Soc..

[90] Aghdam F.A., Rostami A. (2025). In-silico study of lanthanide-based nanoparticles for dual-modal photoacoustic and MRI theranostics. Sci. Rep..

[91] Hu J.-J., Li Y.-G., Wen H.-R., Liu S.-J., Peng Y., Liu C.-M. (2022). Stable Lanthanide Metal–Organic Frameworks with Ratiometric Fluorescence Sensing for Amino Acids and Tunable Proton Conduction and Magnetic Properties. Inorg. Chem..

[92] Zhang X., Tang Z., Song B., Kong D., Yuan J. (2025). Lanthanide Complex-Based Probes for Ratiometric Time-Gated Luminescence and 19F Magnetic Resonance Imaging of Hydrogen Peroxide In Vitro and In Vivo. Anal. Chem..

[93] Liu Q., Ma H., Akam-Baxter E.A., Liu D., Huang Y., Yuan J., Huang H., Song B. (2025). An amphiphilic lanthanide complexes-based liposome nanoprobe for dual-modal time-gated luminescence and magnetic resonance imaging of hypochlorous acid in vitro and in vivo. Chem. Eng. J..

[94] Zhang Y., Li X., Li K., Wang L., Luo X., Zhang Y., Sun N., Zhu M. (2024). DNA binding studies and in-vitro anticancer studies of novel lanthanide complexes. Int. J. Biol. Macromol..

[95] Zhou S., Wang J., Zhang H., Yu W., Geng J. (2025). Constructing a multifunctional cyclic peptide drug delivery platform for gastric cancer nursing therapy. Mat. Lett..

[96] Wang M., He H., Liu D., Ma M., Zhang Y. (2022). Preparation, Characterization and Multiple Biological Properties of Peptide-Modified Cerium Oxide Nanoparticles. Biomolecules.

[97] Luo Z., Yi Z., Liu X. (2023). Surface Engineering of Lanthanide Nanoparticles for Oncotherapy. Acc. Chem. Res..

[98] Calisti L., Trabuco M.C., Boffi A., Testi C., Montemiglio L.C., des Georges A., Benni I., Ilari A., Taciak B., Białasek M. (2018). Engineered ferritin for lanthanide binding. PLoS ONE.

[99] Matsia S., Papadopoulos A., Hatzidimitriou A., Schumacher L., Koldemir A., Pöttgen R., Panagiotopoulou A., Chasapis C.T., Salifoglou A. (2025). Hybrid Lanthanide Metal–Organic Compounds with Flavonoids: Magneto-Optical Properties and Biological Activity Profiles. Int. J. Mol. Sci..

[100] Constantin M., Chioncel M.F., Petrescu L., Vrancianu C.O., Paun M., Cristian R.-E., Sidoroff M., Dionisie M.V., Chifiriuc M.C. (2025). From rock to living systems: Lanthanides toxicity and biological interactions. Ecotoxicol. Environ. Saf..

[101] Pallares R.M., An D.D., Hébert S., Loguinov A., Proctor M., Villalobos J.A., Bjornstad K.A., Rosen C.J., Vulpe C.D., Abergel R.J. (2022). Identifying Toxicity Mechanisms Associated with Early Lanthanide Exposure through Multidimensional Genome-Wide Screening. ACS Omega.

[102] Cawthray J.F., Weekes D.M., Sivak O., Creagh A.L., Ibrahim F., Iafrate M., Haynes C.A., Wasan K.M., Orvig C. (2015). In vivo study and thermodynamic investigation of two lanthanum complexes, La(dpp)3 and La(XT), for the treatment of bone resorption disorders. Chem. Sci..

[103] Scognamiglio P.L., Tesauro D., Roviello G.N. (2025). Metallogels as Supramolecular Platforms for Biomedical Applications: A Review. Processes.

[104] Sargsyan T., Simonyan H.M., Stepanyan L., Tsaturyan A., Vicidomini C., Pastore R., Guerra G., Roviello G.N. (2025). Neuroprotective Properties of Clove (*Syzygium aromaticum*): State of the Art and Future Pharmaceutical Applications for Alzheimer’s Disease. Biomolecules.

[105] Sargsyan T., Stepanyan L., Tsaturyan A., Palumbo R., Vicidomini C., Roviello G.N. (2025). Intracellular Parasitic Infections Caused by *Plasmodium falciparum*, *Leishmania* spp., *Toxoplasma gondii*, *Echinococcus multilocularis*, Among Key Pathogens: Global Burden, Transmission Dynamics, and Vaccine Advances—A Narrative Review with Contextual Insights from Armenia. Vaccines.

[106] Costanzo M., Roviello G.N. (2025). Precision Therapeutics Through Bioactive Compounds: Metabolic Reprogramming, Omics Integration, and Drug Repurposing Strategies. Int. J. Mol. Sci..

[107] Vicidomini C., Roviello G.N. (2025). Therapeutic Convergence in Neurodegeneration: Natural Products, Drug Repurposing, and Biomolecular Targets. Biomolecules.

[108] Sargsyan T., Stepanyan L., Panosyan H., Hakobyan H., Israyelyan M., Tsaturyan A., Hovhannisyan N., Vicidomini C., Mkrtchyan A., Saghyan A. (2025). Synthesis and antifungal activity of Fmoc-protected 1, 2, 4-triazolyl-α-amino acids and their dipeptides against Aspergillus species. Biomolecules.

[109] Ferrara B.T., Thompson E.P., Roviello G.N., Gale T.F. (2025). C-Terminal Analogues of Camostat Retain TMPRSS2 Protease Inhibition: New Synthetic Directions for Antiviral Repurposing of Guanidinium-Based Drugs in Respiratory Infections. Int. J. Mol. Sci..

[110] Sargsyan T., Hakobyan H., Simonyan H., Soghomonyan T., Tsaturyan A., Hovhannisyan A., Sardaryan S., Saghyan A., Roviello G.N. (2025). Biomacromolecular interactions and antioxidant properties of novel synthetic amino acids targeting DNA and serum albumin. J. Mol. Liq..

[111] Stepanyan L., Sargsyan T., Mittova V., Tsetskhladze Z.R., Motsonelidze N., Gorgoshidze E., Nova N., Israyelyan M., Simonyan H., Bisceglie F. (2025). The Synthesis, Characterization, and Biological Evaluation of a Fluorenyl-Methoxycarbonyl-Containing Thioxo-Triazole-Bearing Dipeptide: Antioxidant, Antimicrobial, and BSA/DNA Binding Studies for Potential Therapeutic Applications in ROS Scavenging and Drug Transport. Biomolecules.

[112] Simonyan H., Palumbo R., Vicidomini C., Scognamiglio P.L., Petrosyan S., Sahakyan L., Melikyan G., Saghyan A., Roviello G.N. (2025). Binding of G-quadruplex DNA and serum albumins by synthetic non-proteinogenic amino acids: Implications for c-Myc-related anticancer activity and drug delivery. Mol. Ther. Nucleic Acids.

[113] Hayriyan L., Grigoryan A., Gevorgyan H., Tsaturyan A., Sargsyan A., Langer P., Saghyan A., Mkrtchyan A. (2025). A3-Mannich coupling reaction via chiral propargylglycine Ni(ii) complex: An approach for synthesizing enantiomerically enriched unnatural α-amino acids. RSC Adv..

[114] Tovmasyan A.S., Mkrtchyan A.F., Tsaturyan A.H., Langer P., Malkov A.V., Saghyan A.S. (2025). Strategy for synthesizing O-protected (S)-α-substituted serine analogs via sequential Ni(ii)-complex-mediated cross-coupling and cycloaddition reactions. New J. Chem..

[115] Dadayan A.S., Mkrtchyan A.F., Poghosyan A.S., Dadayan S.A., Stepanyan L.A., Israyelyan M.H., Tovmasyan A.S., Tsaturyan A.H., Hovhannisyan N.A., Topuzyan V.O. (2024). Unnatural Phosphorus-Containing α-Amino Acids and Their N-FMOC Derivatives: Synthesis and In Vitro Investigation of Anticholinesterase Activity. ChemistrySelect.

[116] Tovmasyan A.S., Mkrtchyan A.F., Khachatryan H.N., Hayrapetyan M.V., Hakobyan R.M., Poghosyan A.S., Tsaturyan A.H., Minasyan E.V., Maleev V.I., Larionov V.A. (2023). Synthesis, Characterization, and Study of Catalytic Activity of Chiral Cu(II) and Ni(II) Salen Complexes in the α-Amino Acid C-α Alkylation Reaction. Molecules.

[117] Mkrtchyan A.F., Hayriyan L.A., Karapetyan A.J., Tovmasyan A.S., Tsaturyan A.H., Khrustalev V.N., Maleev V.I., Saghyan A.S. (2020). Using the Ni-[(benzylprolyl)amino]benzophenone complex in the Glaser reaction for the synthesis of bis α-amino acids. New J. Chem..

[118] Mkrtchyan A.F., Saghyan A.S., Hayriyan L.A., Sargsyan A.S., Karapetyan A.J., Tovmasyan A.S., Tsaturyan A.H., Minasyan E.V., Poghosyan A.S., Paloyan A.M. (2020). Asymmetric synthesis, biological activity and molecular docking studies of some unsaturated α-amino acids, derivatives of glycine, allylglycine and propargylglycine. J. Mol. Struct..

[119] Parpart S., Petrosyan A., Ali Shah S.J., Adewale R.A., Ehlers P., Grigoryan T., Mkrtchyan A.F., Mardiyan Z.Z., Karapetyan A.J., Tsaturyan A.H. (2015). Synthesis of optically pure (S)-2-amino-5-arylpent-4-ynoic acids by Sonogashira reactions and their potential use as highly selective potent inhibitors of aldose reductase. RSC Adv..

[120] Vicidomini C., Fontanella F., D’Alessandro T., Roviello G.N., De Stefano C., Stocchi F., Quarantelli M., De Pandis M.F. (2025). Resting-state functional MRI metrics to detect freezing of gait in Parkinson’s disease: A machine learning approach. Comput. Biol. Med..

[121] Roviello G.N. (2025). Nature-Inspired Pathogen and Cancer Protein Covalent Inhibitors: From Plants and Other Natural Sources to Drug Development. Pathogens.

[122] Yi Z., Luo Z., Qin X., Chen Q., Liu X. (2020). Lanthanide-Activated Nanoparticles: A Toolbox for Bioimaging, Therapeutics, and Neuromodulation. Acc. Chem. Res..

